# Immunoproteomic Identification of *Scedosporium boydii* Antigens with Potential Diagnostic Interest in Cystic Fibrosis Patients

**DOI:** 10.1021/acs.jproteome.5c00260

**Published:** 2025-09-25

**Authors:** Leire Martin-Souto, Lucia Abio-Dorronsoro, Maialen Areitio, Leire Aparicio-Fernandez, Oier Rodriguez-Erenaga, Maria Teresa Martin-Gomez, Aitziber Antoran, Aitor Rementeria, Idoia Buldain, Andoni Ramirez-Garcia

**Affiliations:** 1 Department of Immunology, Microbiology and Parasitology, Faculty of Science and Technology, 16402 University of the Basque Country (UPV/EHU), Leioa 48940, Spain; 2 Dept. of Immunology, Microbiology and Parasitology, Faculty of Pharmacy, University of the Basque Country (UPV/EHU), Vitoria-Gasteiz 01006, Spain; 3 Microbiology Department, 16810 Vall d’Hebron University Hospital, Barcelona 08035, Spain

**Keywords:** SERPA, cystic fibrosis, *Scedosporium*/*Lomentospora*, LC-MS/MS, immunoproteomics, antigens

## Abstract

Fungal infections caused by *Scedosporium*/*Lomentospora* species are a significant threat to patients with cystic fibrosis (pwCF), ranking as the second most common filamentous fungi in their airways after *Aspergillus*. Current serodiagnostic methods, such as counterimmunoelectrophoresis and ELISA using crude extracts, lack standardization and commercial availability and often show cross-reactivity with other fungi. This study aimed to identify specific *Scedosporium boydii* antigens with potential for serodiagnosis in pwCF. An immunoproteomic approach combining two-dimensional polyacrylamide gel electrophoresis, Western blot, and mass spectrometry was used to analyze the antigenic profile of *S. boydii* against sera from pwCF with positive cultures for *Scedosporium*/*Lomentospora* (Scedo+) and mice intravenously infected with these fungi. We identified 22 proteins specifically recognized by sera from pwCF Scedo+ and mice infected with *S. boydii*, but not by sera from pwCF or mice infected with *Aspergillus* spp. or uninfected controls. These proteins are primarily involved in metabolism and exhibit catalytic activity. The most prevalent antigens were the heat shock protein 70, 6-phosphogluconate dehydrogenase, 3-ketoacyl-CoA thiolase, and phosphoenolpyruvate carboxykinase, recognized by 67–81% of pwCF Scedo + sera with minimal cross-reactivity to negative samples. This work identifies promising candidates for the specific serodiagnosis of *Scedosporium*/*Lomentospora* infections in pwCF. Data are available via ProteomeXchange (PXD053392).

## Graphical abstract

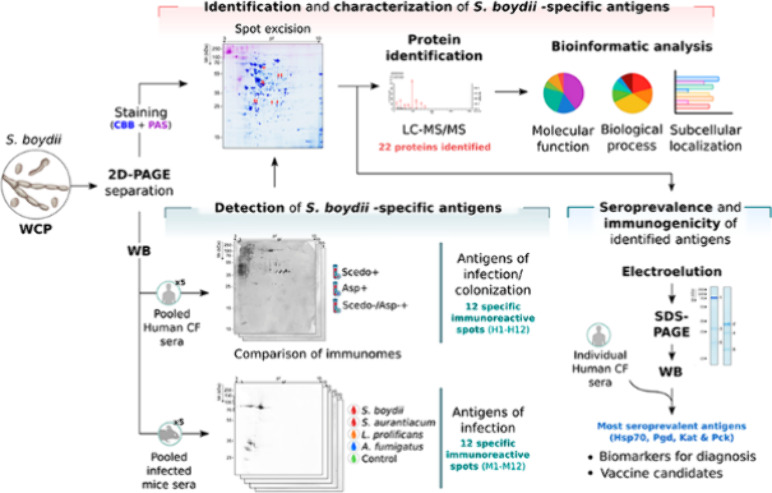

## 1 Introduction

The lack of knowledge, detection strategies, and treatment options for fungal infections has alarmed the World Health Organization (WHO), which has created a list of fungal priority pathogens to guide research, development, and public health action.^1^ Species belonging to the *Scedosporium*/*Lomentospora* genera are among these priority pathogens ranking second, after *Aspergillus*, among the most prevalent filamentous fungi colonizing the respiratory tract of cystic fibrosis (CF) patients that may cause chronic respiratory infections, fungal sensitization or allergic bronchopulmonary mycoses (ABPM).^2^

Measuring serum-specific IgGs is an important tool for the diagnosis of respiratory infections and the differentiation between airway colonization and chronic respiratory infection. The trajectory of *Scedosporium*/*Lomentospora* serology has recently begun by setting up, only in specialized laboratories, Counterimmunoelectrophoresis assays^3^ and Enzyme-Linked Immunosorbent Assay (ELISA) platforms^4^ using crude antigenic extract. Although these techniques are relatively efficient and helpful in detecting *Scedosporium*/*Lomentospora-*positive patients, they are not standardized or commercially available. Moreover, immune cross-reactions due to many polysaccharides and proteins shared with other fungi relevant to the CF lung cannot be disregarded.

Therefore, improvements in serodiagnosis may derive from the use of specific purified antigens. In this respect, based on the well-known biomarkers of *Aspergillus* infection, *Scedosporium* mycelial CatA1 and cytosolic SOD were produced as recombinant antigens, and their value in serodiagnosis was investigated by ELISA.^5^ These purified proteins allowed for the detection of *Scedosporium* infection and the differentiation from an *Aspergillus* infection. Nevertheless, to date, no study has been performed to identify the major specific antigens of *Scedosporium*/*Lomentospora* in a CF context.

To do that, immunoproteomics is a potentially useful tool to identify disease-associated antigens by combining protein separation by two-dimensional polyacrylamide gel electrophoresis (2D-PAGE), immunological detection (immunoblotting), and mass spectrometry. In fact, proteomics-based research, such as serological proteome analysis (SERPA), has been widely used to identify the repertoire of immunoreactive proteins, offering the opportunity to identify and characterize the major antigens of microorganisms, thereby providing a gateway to using them as targets for new and more specific diagnostic methods.

In this sense, this study explores the potential of immunoproteomics to gain insights into the main IgG-reactive and specific antigens of *Scedosporium boydii* whole-cell protein extract (WCP) in a CF context. To do that, sera from patients with CF (pwCF) with and without *Scedosporium*/*Lomentospora,* as well as sera from mice with *Scedosporium*/*Lomentospora* disseminated infection, were employed to detect by Western blot (WB) the most immunoreactive and specific antigens of *S. boydii*, which may be useful for the serological diagnosis of this pathogen.

## 2 Materials and Methods

### 2.1 Human Serum Samples Collection and Categorization

Serum samples from pwCF were obtained from the Vall d’Hebron University Hospital (Barcelona, Spain) and used in this study with the approval of the Ethics Committee from the University of the Basque Country (UPV/EHU) (via material transfer agreement, approval ID: M30/2018/081). Sera were categorized based on the results of the mycological examination of a sputum sample collected in parallel with the sera. Culture conditions and fungal identification were performed as described in a previous study of the group.^6^ According to this, three groups of sera were defined: Group Scedo+ consisted of sera from pwCF with positive cultures for *Scedosporium*/*Lomentospora* spp., group Asp+, pwCF with *Aspergillus* spp. being the only filamentous fungi recovered from sputum; and group Scedo–/Asp– as control consisted of sera from pwCF without any filamentous fungus recovered from samples. Sera were pooled or used individually as indicated.

### 2.2 Serum Samples from Infected Mice

Serum samples from a murine disseminated infection (*Scedosporium boydii*, *Scedosporium aurantiacum*, *Lomentospora prolificans*, and *Aspergillus fumigatus*) were previously obtained by the group^7^ and used in this study. All of the procedures were approved by the Ethics Commission of the UPV/EHU (M20/2016/235, M20/2016/323).

### 2.3 Microorganisms and Culture Conditions

*Scedosporium boydii* CBS 116895 was used in this study. The fungal strain was maintained cryopreserved at −80 °C and cultured as required on potato dextrose agar (PDA) (Condalab, Spain) at 37 °C.

### 2.4 Fungal Proteome Resolution

#### 2.4.1 Obtaining of *S. boydii* Total Whole-Cell Protein Extract (WCP)

A whole-cell protein extract of *S. boydii* was obtained following the protocol optimized by the group.^4^ Proteins were conserved at −20 °C until use.

#### 2.4.2 Two-Dimensional Polyacrylamide Gel Electrophoresis (2D-PAGE)

The precipitation and isoelectric focusing (IEF) of fungal proteins were carried out following the method described by Pellon and co-workers,^8^ but with some modifications in the IEF protocol conditions. 700–800 μg of proteins were loaded into 18 cm long Immobiline DryStrip pH 3–10 or pH 3–6 (GE Healthcare, USA), and IEF was performed at 50 μA per strip and in four steps: step 1, step-and-hold (S&H) mode 500 V for 2000 Vhr; step 2, gradient mode (Gr) 1000 V for 9000 Vhr; step 3; Gr 8000 V for 20000 Vhr; step 4, S&H 8000 V for 250 kVhr. For pH 3–6 strips, the final step 4 was extended for 450 kVhr.

Then, the sodium dodecyl sulfate-polyacrylamide gel electrophoresis (SDS-PAGE) was also performed according to Pellon et al.^8^ on homogeneous 12 and 10% polyacrylamide gels, for pH 3–10 and pH 3–6 strips, respectively. Broad range PageRuler Plus Prestained Protein Ladder 10–250 kDa (Thermo Fisher Scientific, USA) was used as molecular weight standards. 2D-PAGEs were performed in triplicate, and just the most representative gels are shown.

#### 2.4.3 In-Gel Protein Detection by Coomassie Brilliant Blue Staining (CBB)

2D-PAGE gels were stained with Coomassie Brilliant Blue (CBB) G-250 (Sigma-Aldrich, USA)^9^ or CBB R-250 (Sigma-Aldrich) (if used for electroelution) and digitalized using ImageScanner III (GE Healthcare) for immediate protein visualization. Proteome imaging analysis was carried out with ImageMaster 2D Platinum Software (GE Healthcare).

#### 2.4.4 In-Gel glycoprotein Detection by Periodic Acid–Schiff Staining (PAS)

2D-PAGE gels were stained with Schiff’s Reagent (S5133; Sigma-Aldrich) following manufacturer’s instructions. PAS-stained gels were afterward digitalized and analyzed in the same way as CBB gel for image acquisition and analysis.

### 2.5 Antigen Detection by Two-Dimensional Western Blot (2D-WB)

#### 2.5.1 Protein Transfer

2D-PAGE gels were transferred to Amersham Hybond^R^ 0.45 μm PVDF membranes (10600023; Cytiva, UK) at 400 mA for 2 h in the trans-blot semi-dry transfer cell system (Bio-Rad) using Towbin transfer buffer (0.303% [w/v] Tris, 1.44%[w/v] glycine, 20% [v/v] methanol; pH 8.3). Correct transfer was assessed by Ponceau Red stain (0.2% [w/v] Ponceau Red and 1% [v/v] acetic acid).

#### 2.5.2 Immunoblot Using Sera from pwCF and Mice

All of the incubation steps were carried out at room temperature with constant shaking, except when indicated. First, membranes were blocked with tri-*sec*-buffered saline (TBS) containing 0.1% (v/v) Tween 20 and 5% (w/v) skimmed milk powder (TBSTM) for 2 h, and they were incubated overnight at 4 °C with mouse or human pooled sera diluted 1:1000 in TBSTM. The next day, membranes were washed four times, 5 min each, with fresh TBS, and then incubated for 30 min with HRP-labeled antihuman IgG (A6029; Sigma-Aldrich) or antimouse IgG (A9044; Sigma-Aldrich) diluted 1:100,000 in TBSTM. Immunoreactive proteins were detected using the enhanced chemiluminescence substrate kit Amersham ECL Prime (RPN2236; Cytiva, USA) following the manufacturer’s instructions and capturing images after 8 min of exposure in a G:Box Chemi Image acquisition System (Syngene, UK). Membranes were restored with Stripping Buffer (21059; Thermo Fisher Scientific) at 37 °C for 30 min and reprobed with the other pools of sera. All 2D-WB measurements were performed in triplicate.

#### 2.5.3 Oxidation of Glycoproteins

2D membranes were pretreated for 30 min at RT with 50 mM sodium metaperiodate in 100 mM sodium acetate buffer (pH5.5) and washed four times with fresh TBS.

#### 2.5.4 Image Analysis

Analysis of the 2D-WB images was performed using ImageMaster 2D Platinum Software (GE Healthcare). For that, the backgrounds of the 2D-WB images were normalized in reference to those obtained with the Scedo+ group.

### 2.6 Identification of Immunoreactive Proteins by Mass Spectrometry

Immunoreactive antigens meeting the criteria exposed in Table 1 were manually excised from CBB 2D-PAGE gels and subjected to in-gel digestion as described by Buldain and co-workers^10^ Peptides were desalted with homemade C18 tips (3 M Empore C18).

**Table 1:** Antigen Selection Criteria for the LC-MS/MS Identification Process^a^

**process**	**criteria**
LC-MS/MS identification	CF human sera
	Scedo+ specific antigens detected in at least 2 of the 3 replicas with a %Vol >1.4
	nonspecific highly reactive antigens with FC ≥ 4.2 in Scedo+ compared to the immunoreactivity with Asp+ or Scedo–/Asp–
	infected mice sera
	immunoreactive antigens with a %vol > 0.3

a %vol: relative quantification of the antigenic capacity. FC: fold change.

Mass spectrometric analyses were performed on a Q Exactive mass spectrometer interfaced with an EASY-nLC 1000 nanoUPLC system (Thermo Fisher Scientific). Peptides were loaded onto an Acclaim PepMap100 precolumn (75 μm × 2 cm, Thermo Fisher Scientific) connected to an Acclaim PepMap RSLC (50 μm × 15 cm, Thermo Fisher Scientific) analytical column. Peptides were eluted using a linear gradient of 2 to 40% acetonitrile in 0.1% formic acid at a flow rate of 300 nL min^–1^ over 10 min. The mass spectrometer was operated in positive ion mode. Full MS scans (from *m*/*z* 300 to 1850) and MS/MS spectra were acquired with a resolution of 70,000 and 17,500 at *m*/*z* 200, respectively. The 10 most intense ions were fragmented with a normalized collision energy of 28. The maximum ion injection time was 120 ms for both survey and MS/MS scans, whereas AGC target values of 3 × 10^6^ and 5 × 10^5^ were used for survey and MS/MS scans, respectively.

Raw files were processed with Proteome Discoverer 2.2 (Thermo Fisher Scientific) against the NCBInr database (2020–08) restricted to *Scedosporium* (taxonomy ID: 4168, 19693 sequences). For protein identification, the following parameters were adopted: carbamidomethylation of cysteinyl as a fixed modification, oxidation of methionyl as a variable modification, 10 ppm and 0.05 Da for peptide and fragment mass tolerances, and two missed cleavages. Results were filtered for high-confidence peptides and at least three unique peptides. If multiple proteins were found in the same spot, only sequences with an SEQUEST-HT Score exceeding 50% of the highest-scoring protein are displayed. The mass spectrometry proteomics data have been deposited to the ProteomeXchange Consortium via the PRIDE^11^ partner repository with the data set identifier PXD053392

### 2.7 In Silico Analyses of Immunoreactive Proteins

Several online available bioinformatics tools and databases were used to characterize the identified antigens. Table 2 provides a detailed compilation of all the predictive tools employed, including the aim, setup, and threshold values.

**Table 2:** Bioinformatic tools used for antigen characterization^a^

**tool**	**aim**	**threshold (setup)**
NCBI Blastp	protein sequence alignment	N/A
UniProt	general information, biological process, molecular function, and cellular component	N/A
Interpro	classification of protein families, biological process, molecular function, and cellular component	N/A
DeepLoc 2.0	subcellular localization	N/A
SignalP 6.0	standard secretory signal peptides (Sec/SPI)	≥0.5 (Eukarya)
SecretomeP 2.0	nonclassical protein secretion	≥0.6 (mammalian)
Faapred	adhesin-like properties	–0.8 (ACHM model)
AntigenPRO	protein antigenicity prediction	≥0.5
VaxiJen 2.0	protective antigen prediction	≥0.5 (fungal)
AllerTOP v.2	allergenicity prediction	N/A
AllergenFP	allergenicity prediction based on a novel descriptor fingerprint approach	N/A

a Online services and aims with the corresponding likelihood threshold values and default setup parameters. N/A: not applicable.

### 2.8 Purification of Antigens for the Study of Seroprevalence

#### 2.8.1 Electroelution of Selected Antigens

Proteins were electroeluted using a 422 Electro-Eluter system (Bio-Rad), following the manufacturer’s instructions, at 10 mA/chamber (60 mA in total) for 6 h using elution buffer (25 mM Tris base, 192 mM glycine, 0.1% SDS [w/v]). Correct electroelution was assessed by SDS-PAGE and CBB staining, and the protein concentration was estimated by comparison with a BSA standard. Depending on the spot volume, several replicates (between 4 and 10) of the same protein spot were used. Eluted samples were concentrated when needed by using a 100,000 MWCO VIVASPIN centrifugal concentrator system (Sartorius, Germany) and stored at −20 °C until use.

#### 2.8.2 Immunoblot with Human CF Sera

Electroeluted antigens were transferred to PVDF membranes following the protocol described above, but shortening the transfer time to 1 h, and immunoblotted against human CF serum samples individually. The one-dimensional Western blot (1D-WB) was conducted using a MilliBlot-MP membrane processor (Merck Millipore, USA) following the same protocol as the one described for the preceding 2D-WB, except for the dilution of each serum being 1:200 in TBSTM this time. Immunoreactivity was assessed in terms of all-or-nothing (positive or negative) to calculate the antigen recognition prevalence rates.

## 3 Results

### 3.1 Identification of *S. boydii* Antigens Recognized by Serum IgGs from pwCF

The antigenic profile of *S. boydii* WCP was analyzed through an immunoproteomic approach with the purpose of identifying the major specific antigens recognized by serum IgGs from pwCF with positive sputum cultures for *Scedosporium*/*Lomentospora*. *S. boydii* proteome was resolved by 2D-PAGE and CBB gel staining. Up to 1337 blue-dyed protein spots were detected (Figure 1A), 81% of them being smaller than 75 kDa (Figure 2A,D) and 77% of them being located in an isoelectric point (p*I*) range of 4–7 (Figure 2C). PAS staining allowed visualization of the carbohydrate moiety linked to protein cores. Specifically, 147 purple-dyed spots were detected (Figure 1A). Of them, 68% were >75 kDa (Figure 2B,D) and 84% were located in the 3–5 p*I* range (Figure 2C).

**Figure 1:**
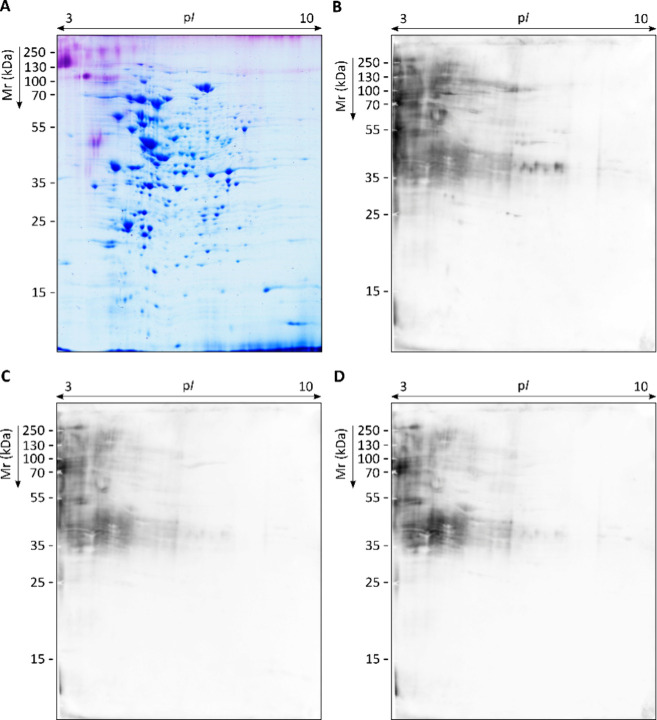
Representative proteome of *S. boydii* and corresponding specific IgG immunoblots using sera pools of pwCF. Total WCP resolved by 2D-PAGE in the p*I* range 3–10 and 12% polyacrylamide gel stained with CBB R-250 and PAS, for protein and glycoprotein visualization, respectively (A). Proteome transferred to PVDF membranes to detect serum IgG-reactive proteins by WB using 1:1000 sera pools of pwCF Scedo+ group (B), Asp + group (C) and Scedo–/Asp– group (D).

**Figure 2:**
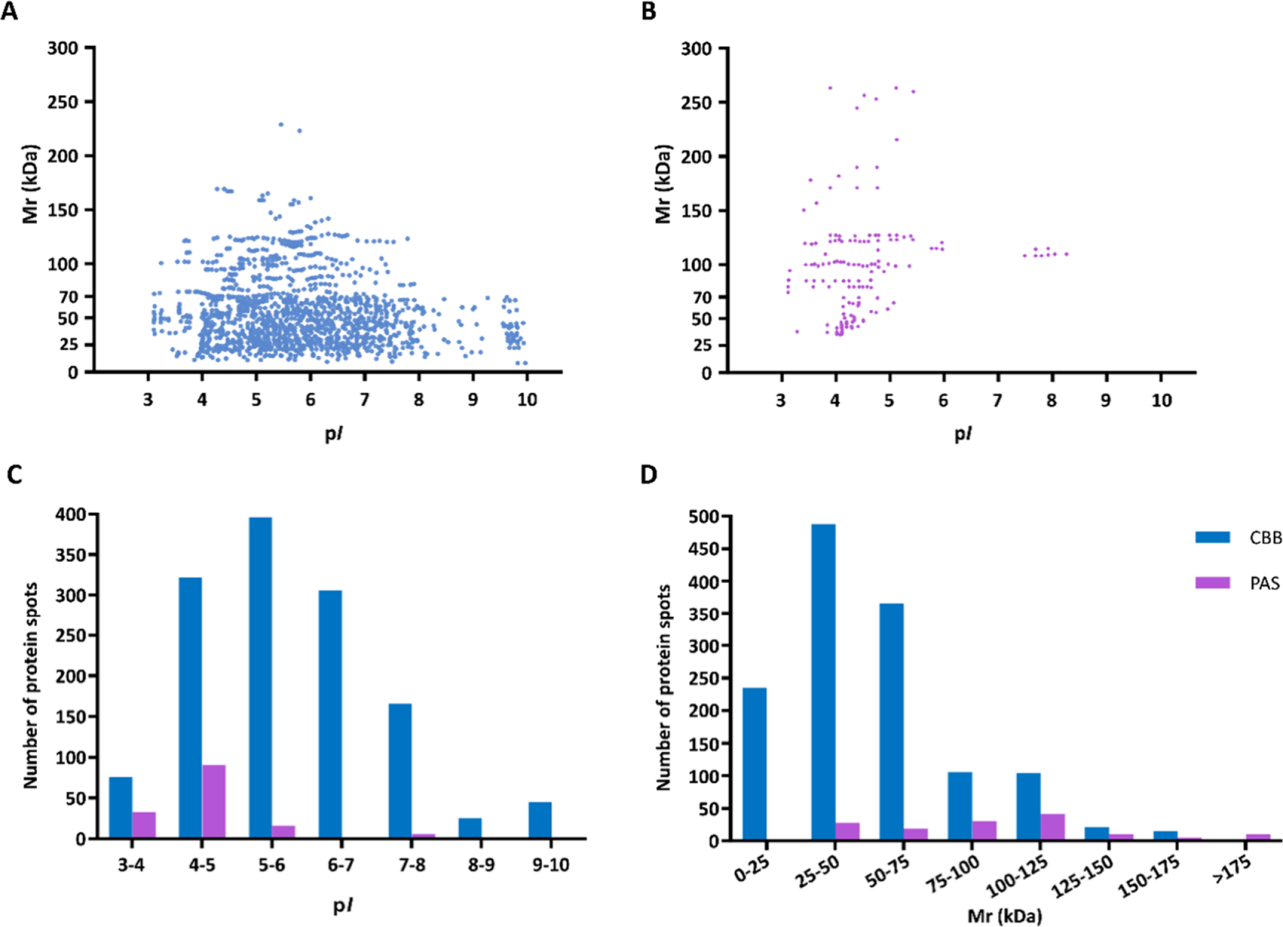
Experimental isoelectric point (p*I*) and molecular mass (*M*_r_) distribution of spots detected in 2D gels after CBB and PAS staining. *M*_r_ and p*I* distributions of protein spots detected in CBB stained 2D gels (A) and glycoproteins detected after PAS staining (B). Frequency of protein spots was based on p*I* (C) and *M*_r_ (D) ranges in CBB and PAS gels.

For antigen detection, immunoblots were performed with three pools of sera from pwCF (Scedo+, Asp+, and Scedo–/Asp−). Regardless of the sera group employed, a blurred and intense reactivity was observed in the upper-left section of the blot (Figure 1B–D). This area corresponded to acidic and high molecular weight (Mr) proteins that were barely visible in the CBB gel but occupied by PAS-stained glycoproteins. This strong fuzzy signal was probably given mainly by carbohydrates and could mask, to some extent, the immunoreactivity of other protein antigens, the latter being of great interest since they may be more specific and easier to produce as recombinant proteins for future diagnostic tests. Thus, membranes were treated with sodium metaperiodate, which oxidizes the carbohydrate fraction of glycoproteins, and WB was repeated. With this treatment, most of the reactivity against fungal glycoproteins disappeared, uncovering many protein antigens with a more defined immunoreactivity (Figure 3B–D).

**Figure 3:**
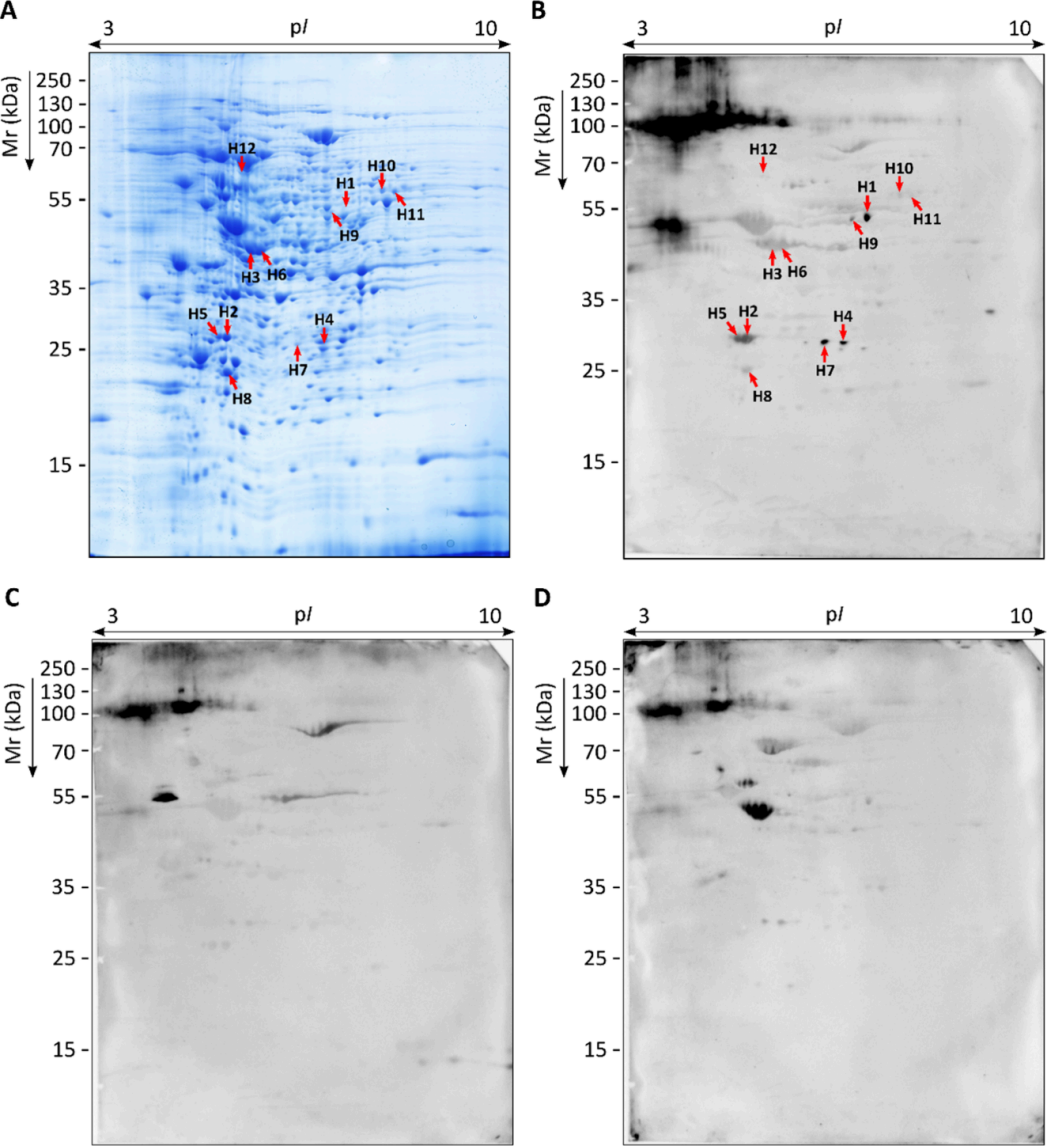
Representative proteome of *S. boydii* and corresponding specific IgG immunoblots using sera pools of pwCF and sodium metaperiodate-oxidized membranes. Total WCP resolved by 2D-PAGE in p*I* range 3–10 and 12% polyacrylamide CBB G-250 stained gel; proteins detected as *Scedosporium*-specific antigens are labeled according to WB images with red arrows (A). Proteome transferred to PVDF membranes to detect serum IgG-reactive proteins by WB using 1:1000 sera pools of pwCF Scedo+ group (B), Asp+ group (C) and Scedo-/Asp- group (D).

From the *S. boydii* proteome (Figure 3A), 132 antigenic spots were found to be recognized by serum IgG from Scedo+ patients (Figure 3B), being scattered on the immunome with *M*_r_ ranging from 21.6 to 110.7 kDa, and a p*I* from 4.9 to 8.7. It is worth noting that some reactivity against noncompletely oxidizes glycoprotein remnants was observed, but this diffuse signal was discarded for the analysis. By comparing this immunome with those obtained using the other two groups of sera pools, 53 antigens were found to be specific for the Scedo+ group (Figure 3C–D). Of the remaining 79 cross-reacting antigens, four were detected overexpressed in Scedo+ immunoblots exhibiting a fold change (FC) of greater than four in comparison to Asp+ or Scedo-/Asp- immunoblots. While not specific, this notable difference in the intensity of reactivity could prove beneficial in future discrimination of these patients.

Using image analysis, the immunoreactive spots (H) meeting the criteria outlined in the “Identification of Immunoreactive Proteins by Mass Spectrometry” subsection of the Materials and Methods were identified by LC-MS/MS. From the 12 spots selected (H1–H12), 17 different proteins were identified (Table 3): 6-phosphogluconate dehydrogenase (Pgd), 40S ribosomal protein S1 (Rps1) and S18 (Rps18), small glutamine-rich tetratricopeptide repeat-containing protein (Sgta), cytochrome c peroxidase (Ccp), mannitol-1-phosphate 5-dehydrogenase (Mpd), l-xylulose reductase (Lxr), tetrahydroxynaphthalene reductase (Thnr), heat shock 70 kDa protein (Hsp70), proteasome subunits alpha (Psma6) and beta (Psmb2), peroxiredoxin-1 (Prdx1), ATP-dependent Clp protease proteolytic subunit (Clpp), 3-ketoacyl-CoA thiolase (Kat), 4-aminobutyrate aminotransferase (Abat), succinyl-CoA:3-ketoacid CoA transferase 1 (Scot), and phosphoenolpyruvate carboxykinase (Pck).

**Table 3:** Identification by LC-MS/MS of Immunodominant Antigens of *S. boydii* total WCP Reacting with Serum IgG from pwCF with *Scedosporium*/*Lomentospora* Positive Cultures^a^

**spot no.**	**accession no.**	**description**	**abbrev.**	**org.**	**uniq. peptid.**	**cover. (%)**	**SEQUEST-HT score**	**theor. p** *I* **/** *M*_**r**_ **(kDa)**	**exp. p** *I* **/** *M*_**r**_ **(kDa)**	**%vol**
H1	XP_016644182.1	6-phosphogluconate dehydrogenase (NAD(+) dependent, decarboxylating)	Pgd	*Sap*	18	36	131.22	6.9/58	6.95/52.71	14.83
H2	XP_016640044	40S ribosomal protein S1 (hp SAPIO_CDS8739)	Rps1	*Sap*	9	29	36.96	10.1/29.3	5.33/29.27	12.69
	XP_016646619.1	SGTA_dimer domain-containing protein (hp SAPIO_CDS0129)	Sgta	*Sap*	5	18	24.96	5.0/35.2		
	KEZ44632.1	cytochrome c peroxidase, mitochondrial	Ccp	*Sap*	7	21	18.05	8.5/39.8		
H3	KEZ40573.1	mannitol-1-phosphate 5-dehydrogenase	Mpd	*Sap*	19	49	150.4	5.5/42.4	5.59/46.35	8.79
H4	XP_016640639.1	l-xylulose reductase	Lxr	*Sap*	13	49	91.39	6.7/28.7	6.63/28.76	8.05
	AIC82365.1	tetrahydroxynaphthalene reductase	Thnr	*Sb*	14	49	69.33	6.4/30.4		
	XP_016646642.1	heat shock 70 kDa protein	Hsp70	*Sap*	11	16	56.11	5.2/71		
H5	XP_016640044	40S ribosomal protein S1 (hp SAPIO_CDS8739)	Rps1	*Sap*	14	52	94.88	10.1/29.3	5.20/29.30	7.24
H6	KEZ40573.1	mannitol-1-phosphate 5-dehydrogenase	Mpd	*Sap*	19	47	203.42	5.5/42.4	5.69/46.29	6.64
H7	KEZ44297.1	Putative proteasome subunit beta type-2/Proteasome endopeptidase complex	Psmb2	*Sap*	7	25	34.26	7.1/21.3	6.38/28.86	5.48
	XP_016638615.1	Proteasome subunit alpha type-6 (hp SAPIO_CDS10856)	Psma6	*Sap*	6	21	32.27	6.0/27.6		
	AIC82365.1	Tetrahydroxynaphthalene reductase	Thnr	*Sb*	4	19	17.62	6.4/30.4		
H8	XP_016646619.1	SGTA_dimer domain-containing protein (hp SAPIO_CDS0129)	Sgta	*Sap*	8	20	23.92	5.1/35.2	5.33/25.38	2.92
	KEZ42914.1	40S ribosomal protein S18 (hp SAPIO_CDS5355)	Rps18	*Sap*	8	48	19.95	10.8/17.8		
	KEZ43947.1	peroxiredoxin-1	Prdx1	*Sap*	5	28	17.66	5.6/24.2		
	XP_016646393.1	ATP-dependent Clp protease proteolytic subunit (hp SAPIO_CDS0406)	Clpp	*Sap*	6	19	16.42	7.8/26.8		
	AIC82365.1	tetrahydroxynaphthalene reductase	Thnr	*Sb*	5	18	12.54	6.4/30.4		
H9	XP_016644182.1	6-phosphogluconate dehydrogenase (NAD(+)-dependent, decarboxylating)	Pgd	Sap	14	27	92	6.95/58	6.75/52.25	2.88
	KEZ45751.1	3-ketoacyl-CoA thiolase, peroxisomal	Kat	*Sap*	11	31	55.03	7.66/44		
H10	XP_016644619.1	4-aminobutyrate aminotransferase	Abat	*Sap*	14	27	102.34	8.79/57.5	7.40/59.28	1.98
	KEZ43571.1	succinyl-CoA:3-ketoacid CoA transferase 1, mitochondrial	Scot	*Sap*	14	25	55.21	7.4/55.2		
H11	XP_016644619.1	4-aminobutyrate aminotransferase	Abat	*Sap*	8	17	23.22	8.8/57.5	7.56/59.11	1.54
H12	XP_016638653.1	phosphoenolpyruvate carboxykinase (ATP)	Pck	*Sap*	20	38	116.12	5.83/64.1	5.56/65.56	1.41

a Accession numbers are reported with protein description and abbreviation. The relative quantification of the spot density is expressed by %vol as a measurement of the spot antigenic capacity with reference to 2D-WB images. Protein experimental (exp.) and theoretical (theor.) molecular weight (*M*_r_) and isoelectric point (p*I*) are reported along with the identification score, sequence coverage percentages (cover.), the number of matching unique peptides (uniq. peptid.), and the organism (org.): *S. apiospermum* (Sap) and *S. boydii* (Sb). Hp: hypothetical protein.

In some spots, more than one protein was identified (Table 3). Likewise, in some cases, the same protein was detected in different spots. All the identified identical proteins are detected in spots with nearly the same *M*_r_ but different p*I*, suggesting that they were isoforms of the same protein, except for proteins on the H8 spot.

### 3.2 In Silico Study of the *S. boydii* Antigens Recognized by Sera from pwCF with *Scedosporium*/*Lomentospora*

Proteins identified by LC-MS/MS were subjected to bioinformatic analyses to study their role and localization in fungal cells. First, functionality of each antigen was studied, and predominantly, they were involved in metabolic processes (*n* = 14; 82.35%), mostly in processes related with protein and carbohydrate metabolism, and to a lesser extent, in response to stress (*n* = 3; 17.65%) and cellular processes regarding protein folding (*n* = 1; 5.8%) (Figure 4A). Regarding their molecular function (Figure 4B), proteins employing catalytic activity were the most abundant (*n* = 14; 82.35%), specifically those with oxidoreductase activity. Likewise, proteins with different binding functions were also prevalent (*n* = 7; 41.18%).

**Figure 4:**
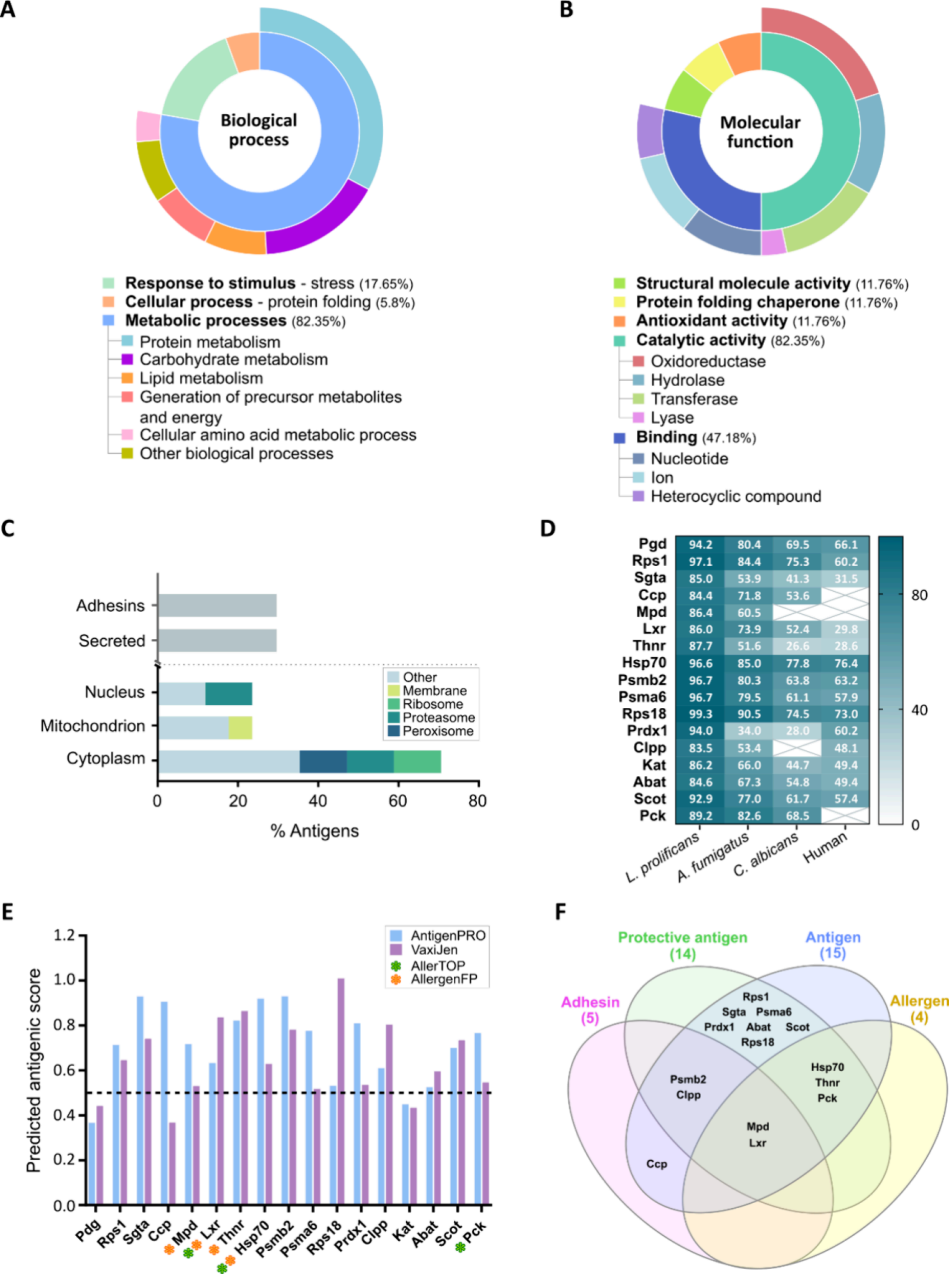
Bioinformatic analysis of *S. boydii* specific antigens recognized by serum IgGs from pwCF. Gene ontology mapping for main biological processes (A) and molecular functions (B) with the corresponding subcategories. Subcellular localization of antigens, secretion via nonclassic pathways and adhesin-like properties (C). Similarity of protein sequences compared to *L. prolificans*, *A. fumigatus*, *C. albicans*, and human indicates identity percentages. Gray crosses over empty squares indicate that no significant similarity was found using blastp (D). Predicted antigenicity and allergenicity (E). Venn diagram summarizing the in silico prediction of adhesins, protective antigens, antigens and allergens (F).

In addition, regarding subcellular localization, the majority of the proteins were predicted to be located in the cytoplasm (*n* = 12; 70.58%), many of them associated with structures like ribosome, proteasome, or peroxisome, but several antigens were predicted to be localized in the cell nucleus (*n* = 4; 23.53%) or mitochondrion (*n* = 4; 23.53%) (Figure 4C). On the other hand, in none of the identified antigens were standard secretory signal peptides found. Nevertheless, Rps1, Ccp, Mpd, Clpp, and Kat were predicted to be secreted via nonclassical pathways (*n* = 5; 29.41%). Moreover, a bioinformatic prediction of adhesin-like proteins was performed, in which Ccp, Mpd, Lxr, Psmb2, and Clpp were found to have adhesin-like properties (*n* = 5; 29.41%). Tables S1–S3 provide a complete biological, molecular, and subcellular characterization of proteins.

The identity of protein sequences with homologous proteins of *Lomentospora prolificans*, *Aspergillus fumigatus*, *Candida albicans*, and human was also analyzed, and, as expected, all the proteins were almost identical in the closely related fungus *L. prolificans*, but many of them were also highly conserved in the other mentioned fungi (Figure 4D). The lowest identity values were observed when compared with human proteins, although some proteins, like Hsp70 or Rps18, exhibited identity percentages above 70%. Ccp, Mpd, and Pck were the least conserved, with no significant similarity to human proteins.

Finally, the results of the prediction of antigenicity and allergenicity are shown in Figure 4E, and all of the scores are detailed in Table S4. According to them, 15 of the identified proteins were predicted to be antigenic, Sgta, Hsp70, Ccp, and Psmb2, showing the greatest scores. Moreover, 14 are probable protective antigens, Lxr, Thnr, Rps18, and Clpp, showing the highest values. Additionally, Mdp, Lxr, Thnr, Hsp70, and Pck were also predicted as allergens, the latter two being identified by the two servers used. It is worth highlighting that Lxr and Mdp were identified as protective antigens, with high antigenicity scores, as well as allergens showing adhesin-like properties. In addition, the latter was also predicted to be secreted via nonclassical pathways (Figure 4F).

### 3.3 Identification of *S. boydii* Antigens Recognized by Serum IgGs from Infected Mice

Altered specific humoral response of pwCF may be related to chronic colonization of the respiratory tract but not necessarily to true infection. In this sense, with the aim of detecting infection-associated antigens, a murine model of *S. boydii* disseminated infection was developed,^7^ and mice sera were used to specifically identify the most immunoreactive antigens.

The same procedure employed for the study of human CF IgG-reactive antigens was now followed with mouse sera, but interestingly, murine IgG-reactive antigenic spots were restricted to a narrow p*I* range from 3 to 5.5 and between 15 kDa and 75 kDa (Figure 5A). Hence, 2D-WB was also performed against the fungal proteome resolved in p*I* 3–6 and 10% polyacrylamide, so as to visualize better the immunome distribution (Figure 5B). Immunoblots using sodium metaperiodate-oxidized membranes were also performed. In contrast to human CF humoral response, which was very strong against fungal glycoproteins, murine infection-related IgG response was more specific toward protein spots (Figure 6).

**Figure 5:**
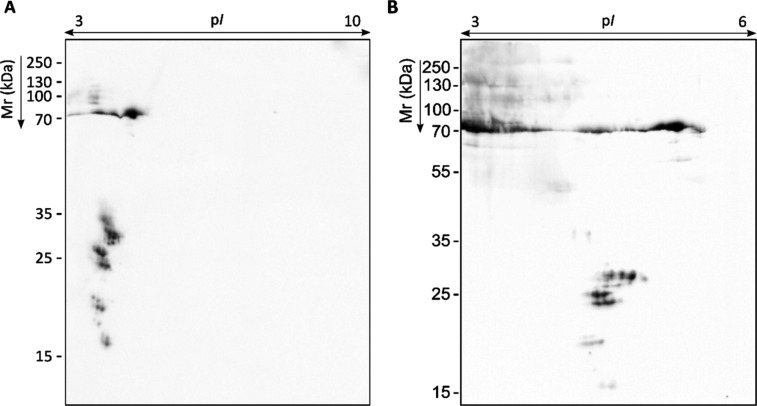
*S. boydii* total WCP immunome recognized by serum IgG from infected mice. Specific IgG immunoblots using 1:1000 pooled sera from mice infected with *S. boydii* against fungal WCP resolved by 2D-PAGE in pI 3–10 and 12% polyacrylamide gel (A), and pI 3–6 and 10% polyacrylamide gel (B).

**Figure 6:**
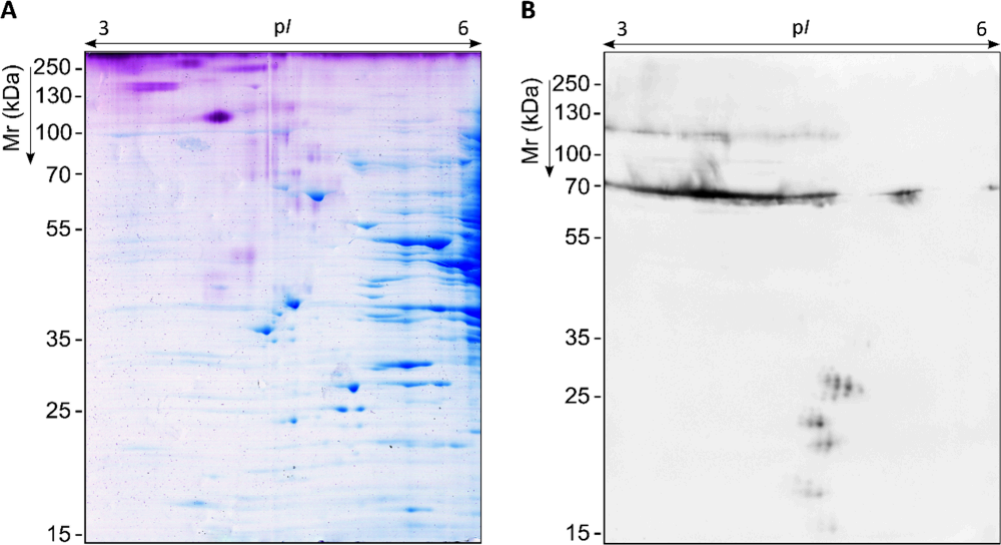
Representative *S. boydii* proteome and corresponding specific IgG immunoblots using pooled sera from infected mice and sodium metaperiodate-oxidized membrane. Total WCP resolved by 2D-PAGE in pI range 3–6 and 10% polyacrylamide gel stained with CBB R-250 and PAS, for protein and glycoprotein visualization, respectively (A). Proteome transferred to PVDF membranes and treated with metaperiodate to detect serum IgG-reactive proteins by WB using 1:1000 sera from mice infected with *S. boydii* (B).

In addition to using sera from mice infected with *S. boydii* (Figure 7), immunoblots were also performed with sera from mice infected with *Scedosporium aurantiacum* (Figure 7C), *L. prolificans* (Figure 7D), or *A. fumigatus* (Figure 7E), as well as sera from the mice control group (Figure 7F). Immunomes of the *Scedosporium/Lomentospora* complex developed an almost identical immunoreactivity pattern (Figure 7B–D). Conversely, mice infected with *A. fumigatus* recognized with very weak reactivity a few *S. boydii* antigens, which were not detected by mice infected with *Scedosporium*/*Lomentospora* spp. Finally, sera from the control group (injected with PBS instead of fungi) did not react with any antigen (Figure 7F).

**Figure 7:**
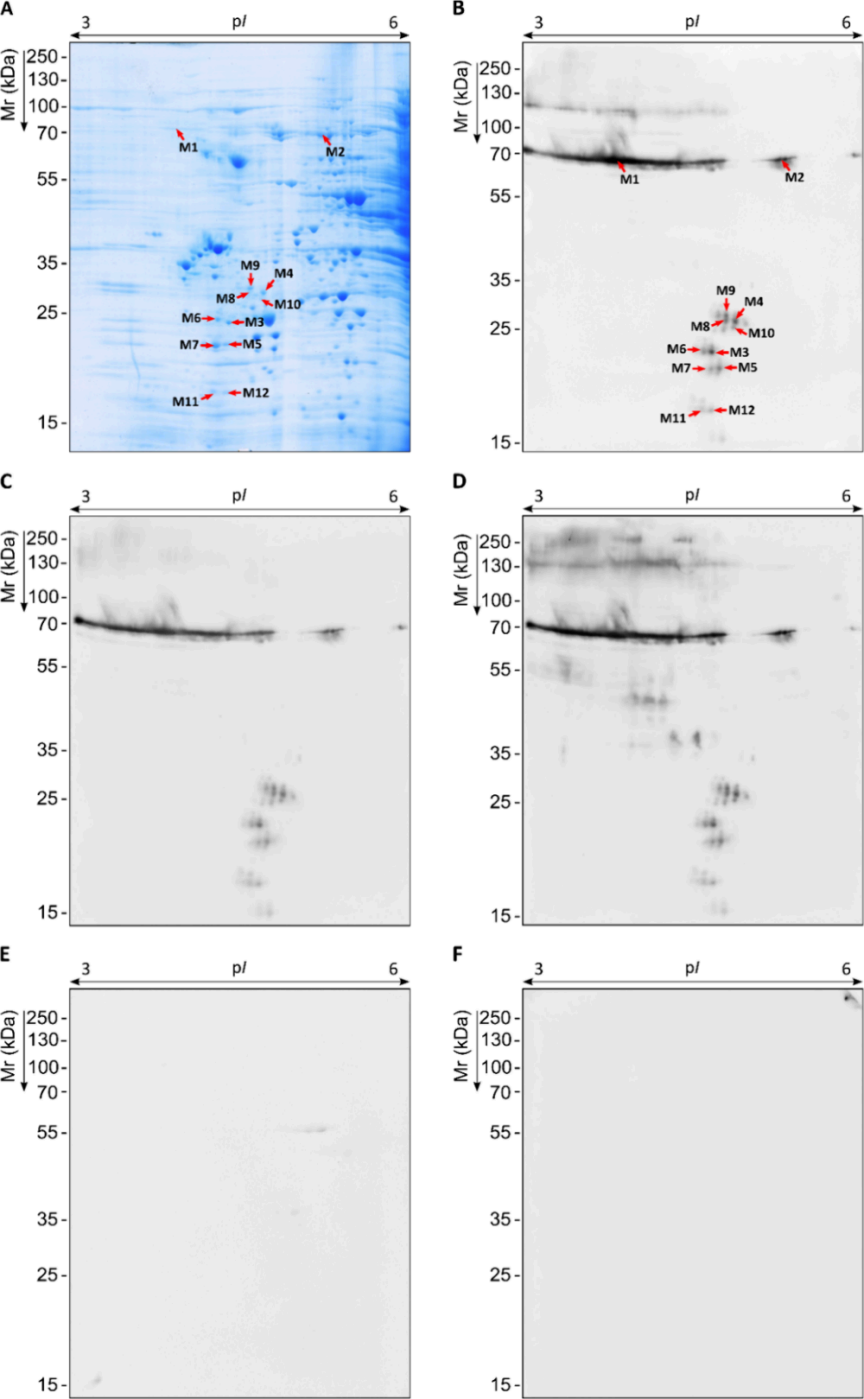
Cross-reactivity study of *S. boydii* antigens using pooled sera from infected mice and sodium metaperiodate-oxidized membranes. Total WCP resolved by 2D-PAGE in p*I* range 3–6 and 10% polyacrylamide CBB G-250 stained gel; proteins detected as *S. boydii*-specific antigens are labeled according to WB images with red arrows (A). Proteome transferred to PVDF membranes to detect serum IgG-reactive proteins by WB using 1:1000 sera pools of mice infected with *S. boydii* (B), *S. aurantiacum* (C), *L. prolificans* (D), *A. fumigatus* (E), and control group (F).

The antigens specifically recognized by mice infected with *S. boydii* (M1-M12) meeting the criteria detailed in Materials and Methods were identified by LC-MS/MS (Table 4) and, surprisingly, spectra obtained from all spots matched with the homologous Hsp70 from *S. apiospermum*genome. However, in three spots (M6, M9, and M10) more than one protein was identified. In these cases, in addition to Hsp70, S5 DRBM domain-containing ribosomal protein (Rps5) and a mitochondrial NFU1 iron–sulfur cluster scaffold-like protein (Nfu1) were identified in spot M6, a 60S ribosomal protein L8 (Rpsl8) was identified in spot M9, and a phosphomannomutase (Pmm) and a proteasome subunit alpha type-5 protein (Psma5) were identified in M10.

**Table 4:** Identification by LC-MS/MS of Immunodominant Antigens of *S. boydii* Total WCP Reacting with Serum IgG from Infected Mice^a^

**spot no.**	**accession no.**	**description**	**abbrev.**	**org.**	**uniq. peptid.**	**cover. (%)**	**SEQUEST- HT Score**	**theor. p** *I* **/** *M*_**r**_ **(kDa)**	**exp. p** *I* **/** *M*_**r**_ **(kDa)**	**%vol**
M1	XP_016646642.1	heat shock 70 kDa protein	Hsp70	*Sap*	37	62	239.65	5.22/71	4.15/68.47	80.14
M2	XP_016646642.1	heat shock 70 kDa protein	Hsp70	*Sap*	23	29	124.68	5.22/71	5.37/67.72	8.46
M3	XP_016646642.1	heat shock 70 kDa protein	Hsp70	*Sap*	16	26	103.90	5.22/71	4.82/25.74	1.14
M4	XP_016646642.1	heat shock 70 kDa protein	Hsp70	*Sap*	12	24	62.47	5.22/71	5.01/29.73	1.11
M5	XP_016646642.1	heat shock 70 kDa protein	Hsp70	*Sap*	14	22	76.62	5.22/71	4.88/24.15	1.04
M6	XP_016646642.1	heat shock 70 kDa protein	Hsp70	*Sap*	13	19	58.05	5.22/71	4.76/25.83	0.72
	KEZ42406.1	S5 DRBM domain-containing protein (hp SAPIO_CDS5590)	Rps5	*Sap*	11	49	47.14	10.40/27.9		
	KEZ43842.1	NFU1 iron–sulfur cluster scaffold-like protein, mitochondrial	Nfu1	*Sap*	6	32	30.16	5.90/33.5		
M7	XP_016646642.1	heat shock 70 kDa protein	Hsp70	*Sap*	14	16	62.92	5.22/71	4.81/23.96	0.58
M8	XP_016646642.1	heat shock 70 kDa protein	Hsp70	*Sap*	9	12	43.93	5.22/71	4.94/29.77	0.55
M9	XP_016646642.1	heat shock 70 kDa protein	Hsp70	*Sap*	14	21	79.83	5.22/71	4.94/30.66	0.53
	KEZ38829.1	60S ribosomal protein L8 (hp SAPIO_CDS10872)	Rpsl8	*Sap*	11	32	60.79	10.37/29.4		
M10	XP_016646642.1	heat shock 70 kDa protein	Hsp70	*Sap*	9	12	41.35	5.22/71	4.99/28.72	0.42
	KEZ44369.1	phosphomannomutase	Pmm	*Sap*	11	42	36.1	5.20/29.7		
	XP_016640852.1	heat shock 70 kDa protein	Hsp70	*Sap*	7	11	32.01	6.13/72.4		
	KEZ41074.1	proteasome subunit alpha type-5(hp SAPIO_CDS7128)	Psma5	*Sap*	8	43	22.3	4.92/26.9		
M11	XP_016646642.1	heat shock 70 kDa protein	Hsp70	*Sap*	10	17	54.99	5.22/71	4.74/20.10	0.40
M12	XP_016646642.1	heat shock 70 kDa protein	Hsp70	*Sap*	11	18	64.76	5.22/71	4.82/20.04	0.38

a Accession numbers are reported with protein description and abbreviation. The relative quantification of the spot density is expressed by %vol as a measurement of the spot antigenic capacity with reference to 2D-WB images. Protein experimental (exp.) and theoretical (theor.) molecular weight (*M*_r_) and isoelectric point (p*I*) are reported along with the identification score, sequence coverage percentages (cover.), the number of matching unique peptides (uniq. peptid.), and the organism (org.): *S. apiospermum* (Sap) and *S. boydii* (Sb). Hp: hypothetical protein

It is striking that Hsp70 is the most immunoreactive antigen against infected mice sera, while, using human CF sera, only a 28 kDa fraction of the protein was detected. In this sense, the area where Hsp70 is usually located in 2D membranes (p*I* 4.8–5 and 65–70 kDa), was immunoblotted using a more concentrated human Scedo+ CF sera (1:200 instead of 1:1000). This time, the Hsp70 protein was clearly detected (Figure 8).

**Figure 8:**
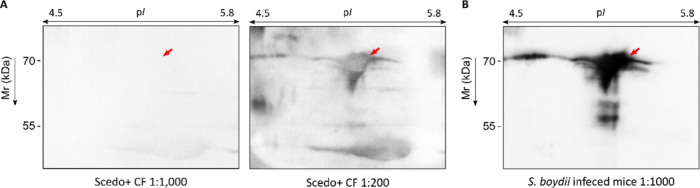
Immunoreactivity of pwCF Scedo+ and infected mice sera against the *S. boydii* Hsp70 region. Immunoblot of Hsp70 area from 2D protein membranes using pwCF Scedo+ sera 1:1,000 and 1:200 (A); and 1:1,000 sera from mice infected with *S. boydii* (B).

### 3.4 In Silico Study of the *S. boydii* Antigens Recognized by Sera from Infected Mice

The bioinformatic analyses of the six proteins identified using sera from mice infected with *S. boydii* showed that metabolic proteins were again the most abundant, with five of them (83.33%) implicated in metabolic processes regarding protein (*n* = 2, 40%), sulfur compounds (*n* = 1, 20%), nucleic acid (*n* = 1, 20%) and nucleotide-sugar metabolisms (*n* = 1, 20%). Two of them were also implicated in cellular processes (33.33%) of protein folding and cellular component assembly. Finally, one of them (16.66%) was involved in stress response, namely Hsp70.

In relation to molecular functions, most of these proteins exhibited binding function (*n* = 4, 66.66%) to nucleotides (*n* = 1, 25%), ions (*n* = 1, 25%) and nucleic acids (*n* = 2, 50%), two of them employed hydrolase and isomerase catalytic activities (*n* = 2, 33.33%), one of them showed structural molecule activity (16.66%), and last, Hsp70 developed a protein folding chaperone function (16.66%).

Predominantly, identified proteins were predicted to be cytosolic (*n* = 5, 83.33%), two of them associated with ribosomes and one with the proteasome core complex. Only Nfu1 was predicted to be located in the mitochondrion and to be secreted via nonconventional pathways. None of the identified proteins were predicted to present adhesin-like properties. Complete information on in silico analyses is detailed in Tables S1–S3.

Looking at protein homology (Figure 9A), sequences were very similar among the indicated species, with Nfu1 being the least conserved one. Concerning the in silico prediction of antigenicity, Hsp70 and Nfu1 achieved the highest scores, although Rps5 and Pmm were also predicted to be antigens. Likewise, five of the identified proteins, Hsp70, Nfu1, Rpsl8, Pmm, and Psma5, were predicted as potential protective antigens, with the latter showing the highest score. Finally, Hsp70, Rps5, and Nfu1 were predicted to be probable allergens, the latter two being identified by both of the servers employed (Figure 9B).

**Figure 9:**
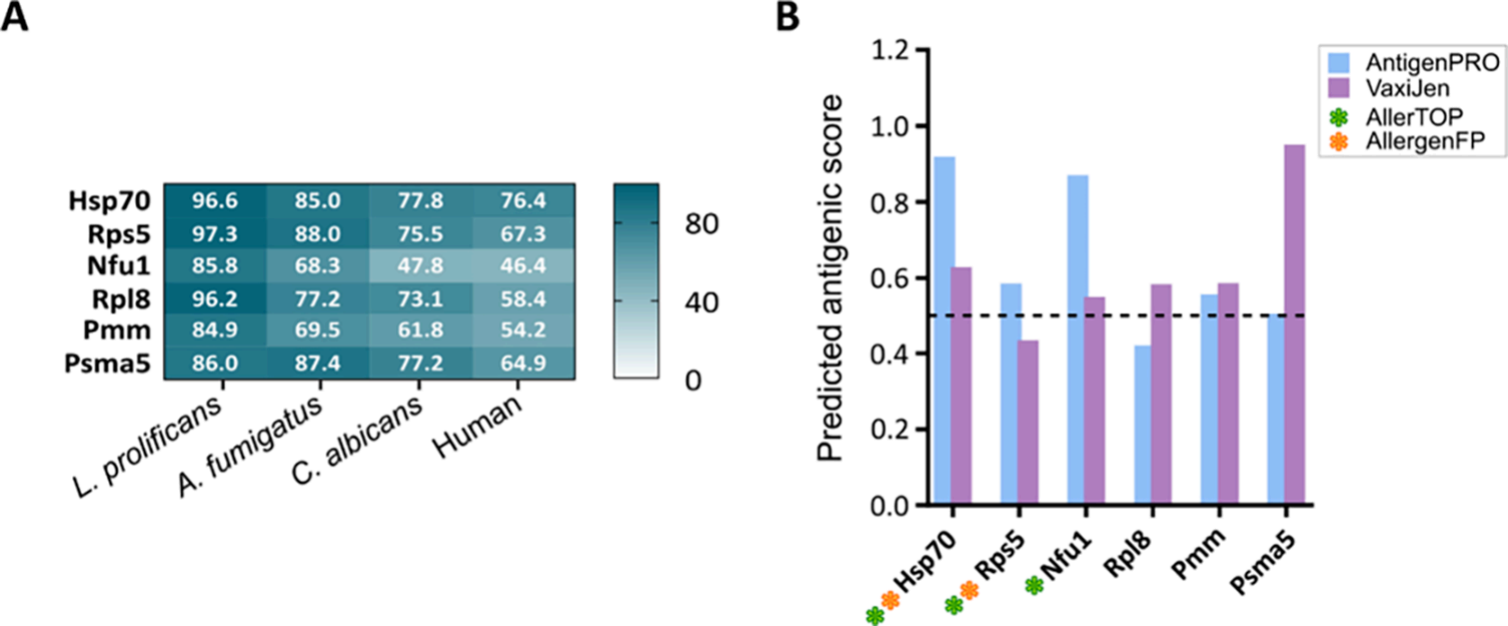
Bioiformatic analysis of LC-MS/MS-identified *S. boydii* proteins reacting with *S. boydii*-infected mice serum IgGs. Similarity of protein sequences compared to *L. prolificans*, *A. fumigatus*, *C. albicans*, and human, indicating identity percentages (A). Predicted antigenicity and allergenicity of the identified proteins. The threshold value for AntigenPRO and VaxiJen is depicted with the dashed line (B).

### 3.5 Seroprevalence and Immunoreactivity of *S. boydii* Antigens

With the aim of studying the utility as diagnostic targets of the antigens identified using both human CF and infected mice sera, the spots with a %vol in protein gels higher than 0.7 were purified from CBB gels by electroelution (Table S5). Some spots were electroeluted together by their proximity to the gel. For M5, M7, M11, and M12, no electroelution was carried out since LC-MS/MS results showed that Hsp70 was the only protein identified, which was correctly isolated during M2 electroelution. Purified proteins were resolved by SDS-PAGE and CBB staining to assess the correct electroelution from gels (Figure 10). All proteins were correctly electroeluted except M3+M6, which were not obtained in a sufficient quantity.

**Figure 10:**
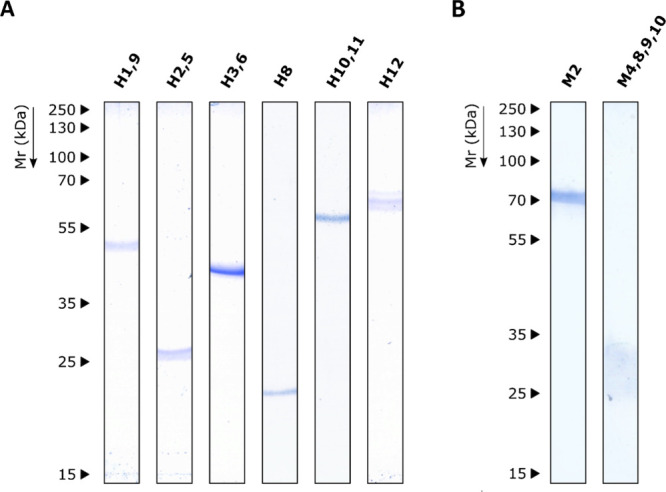
In-gel purification of *S. boydii* antigens. Resolution by SDS-PAGE of electroeluted antigens detected by serum IgG from pwCF with *Scedosporium*/*Lomentospora* (A) and from mice infected with *S. boydii* (B). Each lane corresponds to the electroeluted spots specified above (H for humans and M for mouse).

The antigenic detection rates of the electroeluted proteins were assessed by 1D-WB using human CF serum samples individually. A total of 41 sera were analyzed (21 Scedo+ and 20 Scedo– [10 for Asp+ and 10 for Scedo–/Asp−]) (Figure 11A). As illustrated in Figure 11B, some Scedo+ patients as subjects number 8, 11, 16, and 17 recognized all the antigens electroeluted, whereas, for example, sera from patients 2 or 4 reacted only with H1,9 and M2 antigens.

**Figure 11:**
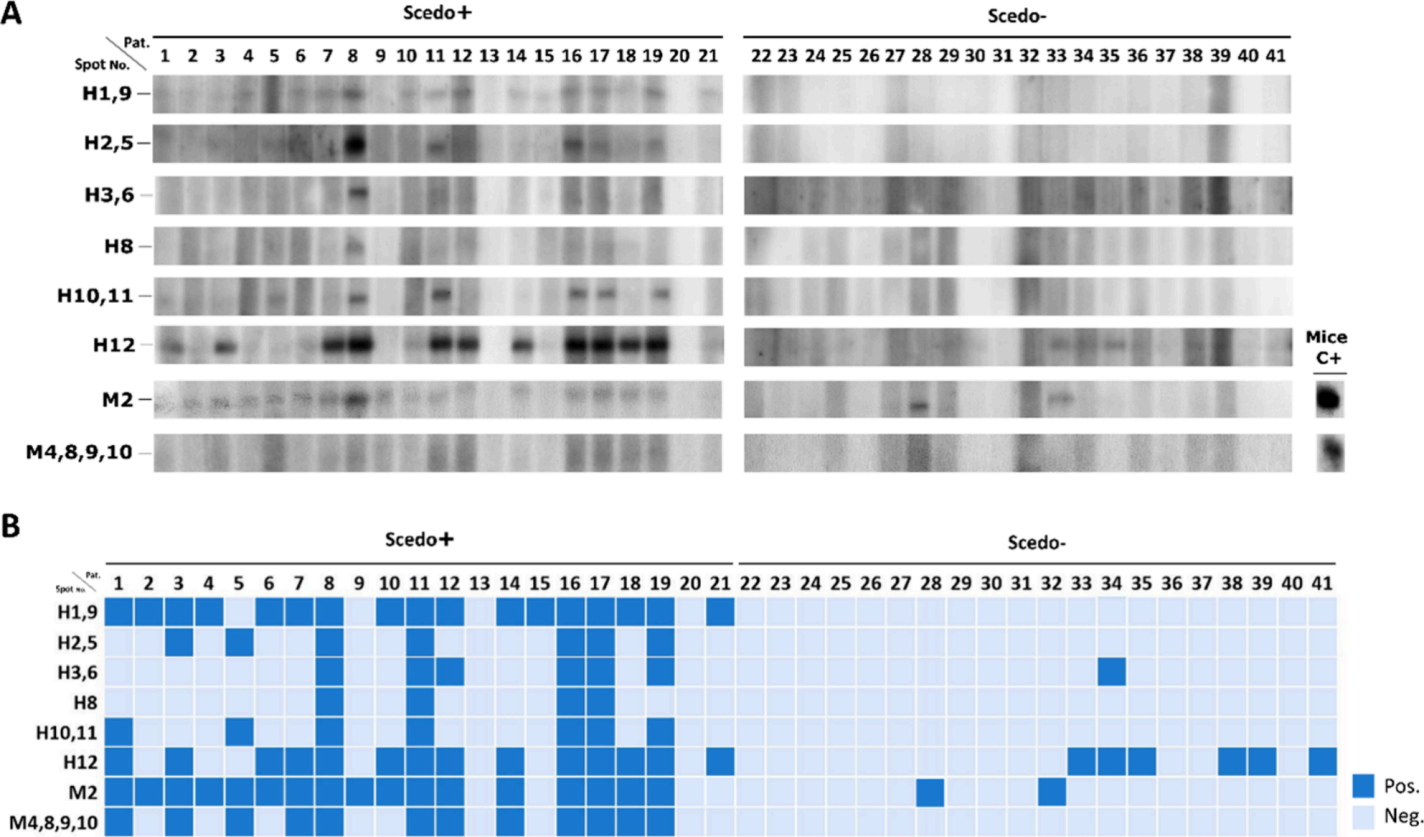
Seroprevalence of *S. boydii* antigens. Antigen recognition prevalence by immunoblotting with individual sera from pwCF. Twenty-one Scedo+ and 20 Scedo– (consisting of 10 Asp+ and 10 Scedo–/Asp−) sera were tested against the electroeluted antigens. 1D-WB images of individual sera (Pat.) and a positive control using infected mice sera (mice C+) (A). Heatmap overview of antigen recognition. Positive result is depicted as a dark blue square and negative as light blue (B). Electroeluted antigens immunoblotted against CF sera are detailed on the left, and the patient number is stated on the top of the charts. In Spot No., H stands for human and M for mouse.

Among the antigens identified from the 2D-WB-based immunoproteomic studies using human sera, H1,9 (Pgd and Kat) and H12 (Pck) antigens were the most prevalent and immunoreactive against Scedo+ samples, being detected by 80.95% and 66.66% of positive sera, respectively. H1,9 was not recognized by any of the negative samples, while H12 was detected by six negative samples with a low intensity. H3,6 (Mpd) showed a prevalence rate of 28.57% within positive sera, H2,5 (Rps1, Sgta, and Ccp) and H10,11 (Abat and Scot) of 33.33%, and H8 (Sgta, Rps1, Prdx1, Cclp, and Thnr) of 19.04%.

Regarding the antigens identified from the study with sera from infected mice, the M2 antigen, corresponding to Hsp70, was the most prevalent within Scedo+ samples (80.95%), being detected only by two negative samples. M4,8,9,10 (Hsp70, Rpsl8, Pmm, and Psma5) were detected by 57.14% of Scedo+ samples but by none of the negative group. Moreover, it is noteworthy that two patients from the Scedo+ group (13 and 20) did not recognize any antigen, so the status of the serum or its correct classification is questionable. If these were removed from the group, then the Hsp70 recognition rate would approach 100% among the positive samples. Recognition prevalence rates of the electroeluted antigens are summarized in Table 5.

**Table 5:** Recognition Prevalence Rates of *S. boydii*-Purified Antigens^a^

**spot** no.	protein	*Scedosporium*+ (*n* = 21)	*Scedosporium*– (*n* = 20)
H1,9	Pgd, Kat	17 (80.95%)	0
H2,5	Rps1, Sgta, Ccp	7 (33.33%)	0
H3,6	Mpd	6 (28.57%)	1 (5%)
H8	Sgta, Rps1, Prdx1, Cclp, Thnr	4 (19.04%)	0
H10,11	Abat, Scot	7 (33.33%)	0
H12	Pck	14 (66.66%)	6 (30%)
M2	Hsp70	17 (80.95%)	2 (10%)
M4,8,9,10	Hsp70, Rpsl8, Pmm, Psma5	12 (57.14%)	0

a The number of sera that recognized the purified antigen (spot no. in 2D gel/WB and identified protein abbreviation) is specified in each case, and the percentage of immunoreactivity events out of the total number of sera tested is stated in brackets. Note that the results of immunoreactivity have been classified in an all-or-none manner without considering differences in intensity. In spot no., H stands for human and M for mouse.

## 4 Discussion

Fungi of the *Scedosporium*/*Lomentospora* genera are of particular importance for pwCF due to the frequency of isolation of these species in their respiratory tract. However, although mycological examination and detection of fungal DNA from respiratory secretions are routinely used to identify these pathogens, they cannot discriminate between chronic colonization of the airways, sensitization of the patient, or respiratory infection, which relies on the detection of *Scedosporium*/*Lomentospora*-specific IgG antibodies. Nevertheless, this serodiagnosis is performed only in highly specialized laboratories using nonstandardized assays and homemade crude antigenic extracts.^12^ Thus, it is crucial to focus efforts on the characterization of new biomarkers.^13^ In this sense, the immunoproteomics-based techniques are very helpful to study host–pathogen interaction-related proteins and to detect the main fungal antigens.^14–17^

In this work, a 2D-WB-based immunoproteomic analysis of *S. boydii* total WCP was performed to identify potential diagnostic targets. To do that, immunoreactivity against pooled sera from pwCF with *Scedosporium*/*Lomentospora* positive cultures (Scedo+) was studied, and the recognition profile was compared to those obtained using *Aspergillus* positive (Asp+) and control CF sera (Scedo–/Asp−) to look for *Scedosporium*-specific antigens.

The first results showed that *S. boydii* glycoproteins were highly immunoreactive, which is consistent with studies carried out in other pathogenic fungi such as **C. albicans*.* ^18^ In this line, glycoproteins have been previously reported as promising novel diagnostic targets,^19^ and, specifically, the peptidorhamnomannan from the mycelium of *S. boydii* was presented as a potential diagnostic antigen.^20,21^ However, the study of glycoproteins presents difficulties such as the fuzzy reactivity in immunoblots, interferences in mass spectrometry identification, or complications for recombinant protein production due to the requirement of an eukaryotic model.^19^ Therefore, in this study, membranes were treated with sodium metaperiodate to oxidize carbohydrate residues and focus the research on protein antigens. In this way, 12 spots that reacted specifically with serum IgGs from Scedo+ patients were detected, which corresponded to 17 different proteins identified by mass spectrometry.

Among them, metabolism-related proteins, mainly involved in protein and carbohydrate metabolic processes, represent the most abundant functional group. These results are in line with those obtained in previous studies performed with the closely related fungus *L. prolificans*.^7,8^ In addition, many of these enzymes are described to exhibit oxidoreductase or hydrolase activity, which is related to the saprophytic metabolism of the fungus and its capacity to degrade and use aliphatic hydrocarbons as a source of carbon and energy.^22^

Specifically, four antigens detected were involved in carbohydrate metabolism. Among them, Mpd is predicted to have adhesin properties and to be secreted, which is in concordance with what other authors describe.^23^ Moreover, this protein is also antigenic in other fungi such as *L. prolificans*,^24^ *Cryptococcus gatti*,^25^ *Neosartorya fischeri*,^26^ *Paracoccidioides lutzii*,^27^ and, in fact, in *A. fumigatus*, showing diagnostic utility allowing the detection of pwCF with allergic bronchopulmonary aspergillosis (ABPA).^26,28^ This antigen has also been related to *A. fumigatus* virulence because of its indirect implication in the quenching of reactive oxygen species (ROS) and temperature stress resistance by mannitol formation.^29,30^ Furthermore, the enzymes Lxr, involved in the eukaryotic pathway of l-arabinose catabolism,^31^ Pgd, involved in evolutionarily conserved central metabolic pathways of the pentose phosphate pathway,^32^ and Pck, the key enzyme of gluconeogenesis,^33^ were also identified. Lxr and Pgd have been previously described as *A. fumigatus* antigens with multiple B-cell and T-cell epitopes, and allergens in ABPA patients, with Pgd even being patent-protected as potentially useful for diagnosis, desensitization therapies, or drug targets.^34–36^ On the contrary, to our knowledge, Pck has not yet been identified as a fungal antigen, although in *Mycobacterium tuberculosis*, it is able to induce T-cell-mediated immune response and is considered a promising new vaccine candidate for tuberculosis.^33^

Regarding protein-metabolism-related proteins, six antigens have been identified. The Clpp is predicted to present adhesin-like properties and to be secreted via nonconventional routes. Clp peptidases target misfolded proteins^37^ and have been described as virulence factors in *Mycobaterium leprae* due to their implication in tissue damage,^38^ and as protective antigens that are part of a divalent vaccine for *Lawsonia intracellularis*.^39^ The enzymes Psma6 and Psmb2, involved in the removal of nonfunctional proteins,^40,41^ have also been identified in this study, and they have been previously described as antigens in other fungi.^7,26,27,34,42^ In general, proteases are believed to play a preeminent role in the development of fungal sensitization during allergies.^43^ Moreover, two structural ribosomal proteins, Rps1 and Rps18, were identified as antigens in this study. They are involved in the cellular translation process and have been reported to contribute to virulence in *C. albicans*, playing important roles in biofilm formation and susceptibility to hydrogen peroxide.^44^ Furthermore, some of these ribosomal proteins have been widely described as antigens of *C. albicans*,^45^ *A. fumigatus* (e.g., Asp f8 and Asp f23 allergens),^34,46,47^ and *L. prolificans*.^10,24^ Although ribosomal proteins are highly conserved, they may be potential vaccines as well as diagnostic markers^48,49^ considering that they are sufficiently divergent to allow species differentiation,^49^ have high antigenicity, and may be relevant in triggering host immune response.^50^ To conclude with the functional group of protein metabolism, the cochaperone Sgta was identified, a protein involved in a variety of cellular functions, including cell cycle control, protein folding, and transport. This protein is described for the first time as a fungal antigen in this study, although it has been identified as a vaccine candidate and diagnostic marker for leishmaniasis,^51^ reporting no cross-reactivity with aspergillosis or paracoccidiomycosis. This fact can be explained by the low homology between these proteins, which was also confirmed in our analysis.

Furthermore, two lipid-metabolism-related proteins were identified: Scot, involved in ketone body catabolic processes, and Kat, a key enzyme in the fatty acid beta-oxidation. Neither of these two proteins has been previously described as an antigen. In addition, Abat, involved in amino acid metabolism and previously reported as an antigen in *A. fumigatus*,^52^ was also detected.

Finally, different proteins related to stress response were also identified. Stress-related proteins help the pathogen to survive under adverse conditions, but many of them are also allergens of *A. fumigatus*.^34,53^ In this study, Prdx1 and Ccp were identified, but while Prdx1 and other peroxiredoxins (e.g., Asp f 3 allergen) have been described as important antigens in *A. fumigatus*,^35,52,54,55^ Ccp has not been described as an antigen so far. However, the probable implication of Ccp in fungal virulence is evident since it is a key enzyme of the oxidative stress response, reducing hydroperoxide, reported in several fungi.^56–60^ Indirectly related to tolerance to oxidative stress, Thnr was also identified. This is a core enzyme of the biosynthetic pathway of dihydroxynaphthalene (DHN)-melanin,^61^ which constitutes the first protective barrier against environmental and host-related stress conditions and drug resistance in dematiaceous fungi. In addition, this protein has been proposed as a diagnostic marker to detect *L. prolificans*.^62^ Lastly, the identification of the Hsp70 completes the set of *S. boydii* antigens specifically recognized by pwCF with *Scedosporium*/*Lomentospora.* However, due to the importance of this protein folding chaperone, previously highlighted by our research group,^7,8,63,64^ it will be discussed further below.

Following the identification of the antigens specifically recognized by sera from pwCF with the presence of *Scedosporium*/*Lomentospora*, this study also aimed to identify infection-associated antigens. This is an important point since current methods do not allow discrimination between colonization and infection, and a positive culture from respiratory samples does not necessarily imply that a true infection is occurring.^65^ For this purpose, sera from mice intravenously infected with different species of the complex, *L. prolificans*, *S. aurantiacum*, and *S. boydii*, as well as mice infected with the prevalent fungus *A. fumigatus*, were immunoblotted against the *S. boydii* proteome to compare the immunomic profiles.

The results obtained suggested that the serological differentiation of species within the *Scedosporium*/*Lomentospora* genera will be very difficult, since the immunomes of these species showed an almost identical immunoreactivity pattern. Nevertheless, discrimination from *Aspergillus* would be quite feasible, as very poor reactivity was observed in the immunoblots performed with sera from mice infected with *A. fumigatus*. This scenario was also described in previous studies performed by our research group with *L. prolificans*.^7^ Interestingly, conversely to what happened with human CF sera, the immunoreactivity of sera from infected mice was highly specific and mainly against protein antigens, since after metaperiodate oxidation of carbohydrate moieties, the reactivity remained almost identical. Among the antigens recognized, the 12 most immunoreactive were identified by mass spectrometry, and surprisingly, the above-mentioned Hsp70 was identified in all of the spots. This protein has been previously proposed as a good diagnostic candidate for *Lomentospora*/*Scedosporium* by our research group^64^ since it was detected as the most immunodominant antigen in the total extract and secretome of *L. prolificans*,^7^ as detected in this study with *S. boydii*. The presence of Hsp70 on the cell surface^8^ and its secretion^7^ make it accessible to the immune response and could explain its high immunogenicity. Moreover, proteins of this family have been previously described as virulence factors^51,66^ and antigens of important pathogenic fungi.^67–69^ Related to heat shock protein (Hsp) family, the Hsp 90 kDa has already been used for the diagnosis of invasive *C. albicans*infections.^70,71^ On the other hand, Hsp70 has been identified as a prevalent antigen of *L. prolificans* recognized by healthy humans,^8,10^ which places this protein in the spotlight because of its prophylactic potential. In this line, Hsps have already been studied as fungal vaccines^72,73^ and even used as adjuvants in vaccines.^74,75^ The weak point of this protein could be the high conservation between species, but the C-terminal fragment of the protein exhibits lower similarity, while maintaining high immunogenicity.^7^ Taking into account that Hsp70 was also identified by sera from pwCF with *Scedosporium*/*Lomentospora*, all of the mentioned information shows the potential of this protein for the development of new diagnostic methods.

Five other different proteins, predominantly metabolism-related and exhibiting binding and catalytic functions, were also identified among the infection-related antigens, in a mixture with Hsp70. Among them, proteasomal and ribosomal proteins were again identified, specifically Psma5, Rpl8, and Rps5. Additionally, Pmm was one of the identified proteins. Although its secretion was not predicted by bioinformatics analysis, this protein was experimentally detected in the secretome of *S. boydii* ^23^ and *P. lutzii*.^76^ It has been previously described as a *C. albicans*antigen^45^ and is involved in the production of mannoprotein-mannose complexes that elicit a cytokine-mediated inflammatory response in *Cryptococcus.* ^25^ An implication in pathogenicity and a possible role of this protein as a target for antifungal drugs has been described in *Cryptococcus neoformans*.^77^ Moreover, it has also been stated to be indispensable for viability, morphogenesis, and cell-wall integrity in *A. fumigatus*.^78^ Eventually, the last protein identified was the mitochondrial Nfu1. This is of great interest since it was predicted to be nonclassically secreted, and along with Hsp70 showed the greatest antigenicity scores. This protein plays an important role in intracellular iron homeostasis^79^ and was previously described as an antigen in *L. prolificans*.^7^ Another stronghold as a diagnostic target is the low homology observed with other pathogenic species and humans.

The differences observed between the antigenic profiles recognized by sera from pwCF and those from infected mice may be due to several factors. In CF patients, *Scedosporium*/*Lomentospora* spp. usually cause chronic colonization of the airways,^2^ while the murine model reflects an acute disseminated infection.^7^ These two clinical contexts are likely to trigger different immune responses. In fact, mice developed a strong systemic reaction predominantly targeting fungal proteins of functional relevance during infection, such as Hsp70, which are involved in stress adaptation and may contribute to virulence. In this acute infection scenario, tissue invasion and fungal clearance driven by a robust immune response increase exposure to these stress response proteins.^64^

In the antigenic prevalence study, the most prevalent serological antigens were Hsp70, Pgd, Kat, and Pck, with 81% of Scedo+ CF sera recognizing the first three and 67% recognizing the latter. Pck was the most immunoreactive antigen in terms of intensity. Moreover, these antigens barely immunoreacted with negative samples, which supports their potential value for diagnosis.

Considering the comprehensive analysis of the *S. boydii* antigens, all of the proteins identified might be good candidates to be studied as novel diagnostic targets, alone or in combination. Nevertheless, the seroprevalence rates observed highlight Hsp70, Pgd, Kat, and Pck as the most promising antigens with potential utility for the serodiagnosis of *Scedosporium*/*Lomentospora* in pwCF. In this sense, the production of these proteins as recombinant antigens, or alternatively, the synthesis of immunogenic peptides of these proteins, are near-future tasks, since this study proves that the novel antigens identified should be considered as candidates to be part of a specific diagnostic test for the detection of a serological response against *Scedosporium* and *Lomentospora* species, and therefore for the differentiation between colonization of the airways and ABPM or respiratory infection in pwCF.

## Supplementary Material



## Notes

(Table S1) Biological process participation of LC-MS/MS-identified proteins; (Table S2) molecular functions of LC-MS/MS-identified proteins; (Table S3) subcellular localization of LC-MS/MS-identified proteins. Gene Ontology reference numbers (GO IDs); (Table S4) prediction of antigenicity; (Table S5) %vol of immunoreactive spots in 2D-PAGE protein gels; original Western blot images (PDF)

## Abbreviations

1D: one-dimensional
2D: two-dimensional
Abat: 4-aminobutyrate aminotransferase
ABPM: allergic bronchopulmonary mycoses
Asp+: pwCF with positive cultures for Aspergillus spp.
CBB: Coomassie Brilliant Blue
Ccp: cytochrome c peroxidase
CF: cystic fibrosis
Clpp: ATP-dependent Clp protease proteolytic subunit
ELISA: enzyme-linked immunosorbent assay
FC: fold change
Gr: gradient mode
Hsp70: heat shock 70 kDa protein
IEF: isoelectric focusing
Kat: 3-ketoacyl-CoA thiolase
LC-MS/MS: liquid chromatography with tandem mass spectrometry
Lxr: l-xylulose reductase
Mpd: mannitol-1-phosphate 5-dehydrogenase
Mr: molecular weight
Nfu1: mitochondrial NFU1 iron–sulfur cluster scaffold-like protein
PAGE: polyacrylamide gel electrophoresis
PAS: periodic acid–Schiff
Pck: phosphoenolpyruvate carboxykinase
PDA: potato dextrose agar
Pgd: 6-phosphogluconate dehydrogenase
p*I*: isoelectric point
Pmm: phosphomannomutase
Prdx1: peroxiredoxin-1
Psma5: proteasome subunit alpha type-5 protein
Psma6: proteasome subunits alpha
Psmb2: proteasome subunits beta
PVDF: poly­(vinylidene fluoride)
pwCF: patients with cystic fibrosis
Rps1: 40S ribosomal protein S1
Rps18: 40S ribosomal protein S18
Rps5: S5 DRBM domain-containing ribosomal protein
Rpsl860S: ribosomal protein L8
S&H: step-and-hold
Scedo-/Asp-: pwCF without any filamentous fungus recovered by culture
Scedo+: pwCF with positive cultures for *Scedosporium*/*Lomentospora*
Scot: succinyl-CoA:3-ketoacid CoA transferase 1
SDS: sodium dodecyl sulfate
SERPA: serological proteome analysis
Sgta: small glutamine-rich tetratricopeptide repeat-containing protein
TBS: Tris-buffered saline
TBSTM: TBS with Tween 20 and skimmed milk powder
Thnr: tetrahydroxynaphthalene reductase
UPV/EHU: University of the Basque Country
WB: Western blot
WCP: whole-cell protein extract
WHO: World Health Organization
