# Supplementary material for: Immunoproteomic Identification of Scedosporium boydii Antigens with Potential Diagnostic Interest in Cystic Fibrosis Patients
Source: J Proteome Res. 2025 Sep 25;24(11):5467–83. doi: 10.1021/acs.jproteome.5c00260 (PMC12604049; doi:10.1021/acs.jproteome.5c00260)
Supplement: Supplementary file 1 [file pr5c00260_si_001.pdf]

SUPPORING INFORMATION FOR PUBLICATION

Immunoproteomic identification of *Scedosporium boydii* antigens with potential diagnostic interest in Cystic Fibrosis patients

Leire Martin-Souto[1], Lucia Abio-Dorronsoro[1], Maialen Areitio[1], Leire Aparicio-Fernandez[1], Oier Rodriguez-Erenaga[1], Maria Teresa Martin-Gomez[3], Aitziber Antoran[1], Aitor Rementeria[1], Idoia Buldain[2] and Andoni Ramirez-Garcia[1].*

[1] Department of Immunology, Microbiology and Parasitology, Faculty of Science and Technology, University of the Basque Country (UPV/EHU), 48940 Leioa, Spain.

[2] Dept. of Immunology, Microbiology and Parasitology, Faculty of Pharmacy, University of the Basque Country (UPV/EHU), 01006 Vitoria-Gasteiz, Spain

[3] Microbiology Department, Vall d'Hebron University Hospital, 08035 Barcelona, Spain

Table of Contents

**Component of supporting information**	**Brief caption**
Table S1	Biological process participation of LC-MS/MS identified proteins
Table S2	Molecular functions of LC-MS/MS identified proteins
Table S3	Subcellular localization of LC-MS/MS identified proteins.
Table S4	Prediction of Antigenicity
Table S5	%Vol of immunoreactive spots in 2D-PAGE protein gels
Figure S1	Uncropped original Western Blot images

Table S1. Biological process participation of LC-MS/MS identified proteins. Gene Ontology reference number **(GO ID).**

Abbrev.	Protein description	General biological process	Specific process	GO ID
**Pgd**	6-phosphogluconate dehydrogenase (NAD(+) dependent, decarboxylating)	Carbohydrate metabolic process	D-gluconate metabolic process	0019521
		Generation of precursor metabolites and energy	Pentose-phosphate shunt	0006098
**Rps1**	40S ribosomal protein S1	Protein cellular and metabolic process	Translation	0006412
**Sgta**	SGTA_dimer domain-containing protein	Protein modification process	Protein modification by small protein conjugation or removal	0070647
**Ccp**	Cytochrome c peroxidase, mitochondrial	Cellular response to oxidative stress	-	0034599
**Mpd**	Mannitol-1-phosphate 5-dehydrogenase	Carbohydrate metabolic process	Mannitol metabolic process	0019594
**Lxr**	L-xylulose reductase	Carbohydrate metabolic process	Xylulose metabolic process	0005997
**Thnr**	Tetrahydroxynaphthalene reductase	Other biological process	Melanin byosinthetic process	0042438
**Hsp70**	Heat shock 70 kDa protein	Protein folding and stress response	-	0006457
**Psmb2**	Putative proteasome subunit beta type-2 / Proteasome endopeptidase complex	Protein catabolic process	Proteolysis involved in cellular protein catabolic process	0051603
			Proteasomal protein catabolic process	0010498
**Psma6**	Proteasome subunit alpha type-6	Protein catabolic process	Ubiquitin-dependent protein catabolic process	0006511
			Proteolysis involved in cellular protein catabolic process	0051603
**Rps18**	40S ribosomal protein S18	Protein metabolic process	Translation	0006412
**Prdx1**	Peroxiredoxin-1	Cellular response to oxidative stress	-	0034599
**Clpp**	ATP-dependent Clp protease proteolytic subunit	Protein metabolic process	Proteolysis	0006508
**Kat**	3-ketoacyl-CoA thiolase, peroxisomal	Lipid metabolic process	Fatty acid beta-oxidation	0006635
		Other biological process	Phenylacetate catabolic process	0010124
**Abat**	4-aminobutyrate aminotransferase	Cellular amino acid metabolic process	Gamma-aminobutyric acid metabolic process	0009448
**Scot**	Succinyl-CoA:3-ketoacid CoA transferase 1, mitocondrial	Lipid metabolic process and generation of precursor metabolites and energy	Ketone body catabolic process	0046952
**Pck**	Phosphoenolpyruvate carboxykinase (ATP)	Carbohydrate metabolic process	Gluconeogenesis	0006094
**Rps5**	S5 DRBM domain-containing protein	Protein cellular and metabolic process	Translation	0006412
**Nfu1**	NFU1 iron-sulfur cluster scaffold-like protein, mitochondrial	Cellular component assembly and sulfur compound metabolic processes	Iron-sulfur cluster assembly	0016226
**Rpl8**	60S ribosomal protein L8	Ribonucleoprotein complex biogenesis and nucleic acid metabolic processes	rRNA processing	0006364
**Pmm**	Phosphomannomutase	Nucleotide-sugar biosynthetic and GDP-mannose metabolic processes	GDP-mannose biosynthetic process	0009298
**Psma5**	Proteasome subunit alpha type-5	Protein catabolic process	Proteasome-mediated ubiquitin-dependent protein catabolic process	0043161
			Proteolysis involved in cellular protein catabolic process	0051603

Table S2. Molecular functions of LC-MS/MS identified proteins. Gene Ontology reference number **(GO ID).**

Abbrev.	Description	General molecular function	Specific function	GO ID
**Pgd**	6-phosphogluconate dehydrogenase (NAD(+) dependent, decarboxylating)	Oxidoreductase activity	Phosphogluconate dehydrogenase (descarboxylating) activity	0004616
		Small molecule binding	NADP binding	0050661
**Rps1**	40S ribosomal protein S1	Structural molecule activity	Structural constituent of ribosome	0003735
**Sgta**	SGTA_dimer domain-containing protein	Metal ion binding	Zinc ion binding	0008270
**Ccp**	Cytochrome c peroxidase, mitochondrial	Antioxidant activity	Peroxidase activity	0004601
		Metal ion binding	-	0046872
		Heme binding	-	0020037
**Mpd**	Mannitol-1-phosphate 5-dehydrogenase	Oxidoreductase activity	Mannitol-1-phosphate 5-dehydrogenase activity	0008926
**Lxr**	L-xylulose reductase	Oxidoreductase activity	L-xylulose reductase (NADP+) activity<br>Sorbose reductase activity	0050038<br>0032115
**Thnr**	Tetrahydroxynaphthalene reductase	Oxidoreductase activity	Tetrahydroxynaphthalene reductase activity	0047039
**Hsp70**	Heat shock 70 kDa protein	Hydrolase activity<br>Protein folding chaperone<br>Small molecule binding	ATP hydrolysis activity<br>ATP -dependent folding protein<br>ATP binding	0016887<br>0140662<br>0005524
**Psmb2**	Putative proteasome subunit beta type-2 / Proteasome endopeptidase complex	Hydrolase activity	Threonine-type endopeptidase activity	0004298
**Psma6**	Proteasome subunit alpha type-6	-	-	-
**Rps18**	40S ribosomal protein S18	Structural molecule activity	Structural constituent of ribosome	0003735
		Nucleic acid binding	RNA binding	0003723
**Prdx1**	Peroxiredoxin-1	Antioxidant activity	Peroxidase activity<br>Peroxidredoxin activity	0004601<br>0051920
**Clpp**	ATP-dependent Clp protease proteolytic subunit	ATP-dependent catalytic activity	ATP-dependent peptidase activity	0004176
		Hydrolase activity	Serine-type endopeptidase activity	0004252
**Kat**	3-ketoacyl-CoA thiolase, peroxisomal	Transferase activity	Acyltransferase activity	0016747
**Abat**	4-aminobutyrate aminotransferase	Transferase activity	4-aminobutyrate: 2-oxoglutarate transaminase activity	0034386
		Small molecule binding	Pyridoxal phosphate binding	0030170
**Scot**	Succinyl-CoA:3-ketoacid CoA transferase 1, mitocondrial	Transferase activity	3-oxoacid CoA-transferase activity	0008260
**Pck**	Phosphoenolpyruvate carboxykinase (ATP)	Transferase activity	Kinase activity	0016301
		Lyase activity	Phosphoenolpyruvate carboxykinase (ATP) activity	0004612
		Small molecule binding	ATP binding	0005524
**Rps5**	S5 DRBM domain-containing protein	Structural molecule activity	Structural constituent of ribosome	0003735
		Nucleic acid binding	RNA binding	0003723
**Nfu1**	NFU1 iron-sulfur cluster scaffold-like protein, mitochondrial	Metal ion binding	Iron ion binding	0005506
		Metal cluster binding	Iron-sulfur cluster binding	0051536
**Rpl8**	60S ribosomal protein L8	Nucleic acid binding	RNA binding	0003723
**Pmm**	Phosphomannomutase	Isomerase catalytic activity	Phosphomannomutase activity	0004615
**Psma5**	Proteasome subunit alpha type-5	-	-	-

Table S3. Subcellular localization of LC-MS/MS identified proteins. Gene Ontology reference number **(GO ID).**

Abbrev.	Description	DeepLoc location	DeepLoc sorting signal	UniProt Localization (GO term)	GO ID
**Pgd**	6-phosphogluconate dehydrogenase (NAD(+) dependent, decarboxylating)	Cytoplasm	Peroxisomal targeting signal	-	-
**Rps1**	40S ribosomal protein S1	Cytoplasm	Nuclear localization signal	(Cytoplasm) Cytosolic small ribosomal subunit	0022627
**Sgta**	SGTA_dimer domain-containing protein	Cytoplasm	Nuclear localization signal	-	-
**Ccp**	Cytochrome c peroxidase, mitochondrial	Mitochondrion	Mitochondrial transit peptide	(Membrane) Integral component of membrane	0016021
**Mpd**	Mannitol-1-phosphate 5-dehydrogenase	Cytoplasm	Nuclear localization signal	-	-
**Lxr**	L-xylulose reductase	Cytoplasm	-	-	-
**Thnr**	Tetrahydroxynaphthalene reductase	Cytoplasm	-	-	-
**Hsp70**	Heat shock 70 kDa protein	Cytoplasm	Nuclear export signal	-	-
**Psmb2**	Putative proteasome subunit beta type-2 / Proteasome endopeptidase complex	Cytoplasm, Nucleus	Nuclear export signal	(Proteasome) Proteasome core complex	0005839
**Psma6**	Proteasome subunit alpha type-6	-	-	(Cytoplasm)(Nucleus) Proteasome core complex, alpha-subunit complex	0019773
**Rps18**	40S ribosomal protein S18	Cytoplasm, Nucleus		Ribosome	0005840
**Prdx1**	Peroxiredoxin-1	Nucleus	Nuclear localization signal	-	-
**Clpp**	ATP-dependent Clp protease proteolytic subunit	Mitochondrion	Mitochondrial transit peptide	-	-
**Kat**	3-ketoacyl-CoA thiolase, peroxisomal	Cytoplasm	Mitochondrial transit peptide	-	-
**Abat**	4-aminobutyrate aminotransferase	Mitochondrion	Mitochondrial transit peptide	-	-
**Scot**	Succinyl-CoA:3-ketoacid CoA transferase 1, mitocondrial	Mitochondrion	Mitochondrial transit peptide	Mitochondrion	0005739
**Pck**	Phosphoenolpyruvate carboxykinase (ATP)	Cytoplasm	-	-	-
**Rps5**	S5 DRBM domain-containing protein	Cytoplasm, Nucleus	Nuclear localization signal	(Ribosome) Small ribosomal subunit	0015935
**Nfu1**	NFU1 iron-sulfur cluster scaffold-like protein, mitochondrial	Mitochondrion	Mitochondrial transit peptide	-	-
**Rpl8**	60S ribosomal protein L8	Cytoplasm	Nuclear localization signal	(Ribosome) Cytosolic large ribosomal subunit	0022625
**Pmm**	Phosphomannomutase	Cytoplasm	-	Cytoplasm	0005737
**Psma5**	Proteasome subunit alpha type-5	Cytoplasm, Nucleus	Nuclear export signal	Proteasome core complex	0005839

Table S4. Prediction of Antigenicity. The antigenicity of the identified proteins was predicted using AntigenPRO, VaxiJen (protective antigenicity), AllerTOP and AllerfenFP servers. Probability values in **bold** crossed the threshold stablished, so they are considered as probable antigens; **A**: predicted as allergen.

Abbrev.	Description	AntigenPRO	VaxiJen	AllerTOP	AllergenFP
Pgd	6-phosphogluconate dehydrogenase (NAD(+) dependent, decarboxylating)	0.3609	0.4364		
Rps1	40S ribosomal protein S1	**0.7064**	**0.6399**		
Sgta	SGTA_dimer domain-containing protein	**0.9235**	**0.7355**		
Ccp	Cytochrome c peroxidase, mitochondrial	**0.8992**	0.3620		
Mpd	Mannitol-1-phosphate 5-dehydrogenase	**0.7103**	**0.5244**		A
Lxr	L-xylulose reductase	**0.6267**	**0.8294**	A	A
Thnr	Tetrahydroxynaphthalene reductase	**0.8157**	**0.8587**		A
Hsp70	Heat shock 70 kDa protein	**0.9134**	**0.6226**	A	A
Psmb2	Putative proteasome subunit beta type-2 / Proteasome endopeptidase complex	**0.9245**	**0.7758**		
Psma6	Proteasome subunit alpha type-6	**0.7702**	**0.5109**		
Rps18	40S ribosomal protein S18	**0.5253**	**1.0031**		
Prdx1	Peroxiredoxin-1	**0.8032**	**0.5298**		
Clpp	ATP-dependent Clp protease proteolytic subunit	**0.6035**	**0.7978**		
Kat	3-ketoacyl-CoA thiolase, peroxisomal	0.4426	0.4285		
Abat	4-aminobutyrate aminotransferase	**0.5189**	**0.5905**		
Scot	Succinyl-CoA:3-ketoacid CoA transferase 1, mitocondrial	**0.6943**	**0.7286**		
Pck	Phosphoenolpyruvate carboxykinase (ATP)	**0.7598**	**0.5408**	A	
Rps5	S5 DRBM domain-containing protein	**0.5798**	0.4284	A	A
Nfu1	NFU1 iron-sulfur cluster scaffold-like protein, mitochondrial	**0.8648**	**0.5447**	A	
Rpl8	60S ribosomal protein L8	0.4154	**0.5778**		
Pmm	Phosphomannomutase	**0.5513**	**0.5808**		
Psma5	Proteasome subunit alpha type-5	0.5000	**0.9462**		

Table S5. %Vol of immunoreactive spots in 2D-PAGE protein gels.

Gel	Spot No.	%Vol	Gel	Spot No.	%Vol
**pI 3-10, 12%**	H1	1.17	**pI 3-6, 10%**	M1	8.98
	H2	18.72		M2	31.13
	H3	24.40		M3	8.79
	H4	0.61		M4	10.65
	H5	7.84		M5	1.73
	H6	24.66		M6	3.08
	H7	0.36		M7	20.16
	H8	13.07		M8	0.90
	H9	0.79		M9	8.50
	H10	1.50		M10	1.72
	H11	3.61		M11	3.16
	H12	3.28		M12	1.18

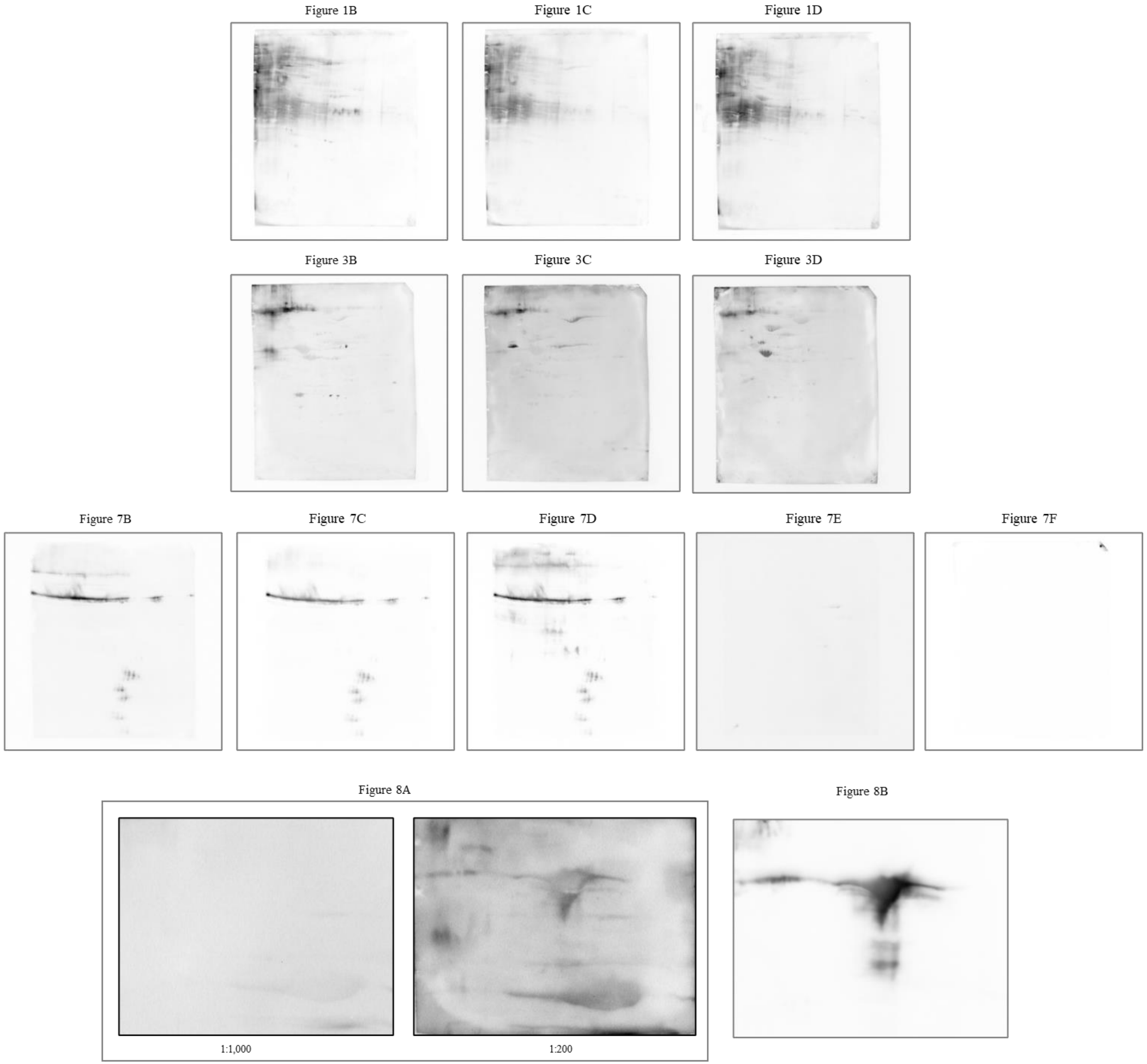

Figure S1. Uncropped original Western Blot images.
